# The urinary excretion of epigenetically modified DNA as a marker of pediatric ALL status and chemotherapy response

**DOI:** 10.1038/s41598-021-00880-9

**Published:** 2021-11-01

**Authors:** Rafal Rozalski, Daniel Gackowski, Aleksandra Skalska-Bugala, Marta Starczak, Agnieszka Siomek-Gorecka, Ewelina Zarakowska, Martyna Modrzejewska, Tomasz Dziaman, Anna Szpila, Kinga Linowiecka, Jolanta Guz, Justyna Szpotan, Maciej Gawronski, Anna Labejszo, Lidia Gackowska, Marek Foksinski, Elwira Olinska, Aleksandra Wasilow, Andrzej Koltan, Jan Styczynski, Ryszard Olinski

**Affiliations:** 1 Department of Clinical Biochemistry, Faculty of Pharmacy, Collegium Medicum in Bydgoszcz, Nicolaus Copernicus University in Toruń, 85–092 Bydgoszcz, Poland; 2 Department of Human Biology, Institute of Biology, Faculty of Biological and Veterinary Sciences, Nicolaus Copernicus University in Toruń, 87–100 Toruń, Poland; 3 Department of Immunology, Faculty of Pharmacy, Collegium Medicum in Bydgoszcz, Nicolaus Copernicus University in Toruń, 85–092 Bydgoszcz, Poland; 4 District Health Center in Kartuzy, 83–300 Kartuzy, Poland; 5 Department of Pediatric, Hematology and Oncology, Collegium Medicum in Bydgoszcz, Nicolaus Copernicus University in Toruń, 85–092 Bydgoszcz, Poland; 6 Department of Geriatrics, Division of Biochemistry and Biogerontology, Collegium Medicum in Bydgoszcz, Nicolaus Copernicus University in Toruń, 85–092 Bydgoszcz, Poland

**Keywords:** DNA, Biomarkers, Haematological diseases, Paediatric cancer

## Abstract

The active DNA demethylation process may be linked to aberrant methylation and may be involved in leukemogenesis. We investigated the role of epigenetic DNA modifications in childhood acute lymphoblastic leukemia (ALL) diagnostics and therapy monitoring. We analyzed the levels of 5-methyl-2′-deoxycytidine (5-mdC) oxidation products in the cellular DNA and urine of children with ALL (at diagnosis and during chemotherapy, n = 55) using two-dimensional ultra-performance liquid chromatography with tandem mass spectrometry (2D UPLC–MS/MS). Moreover, the expression of Ten Eleven Translocation enzymes (TETs) at the mRNA and protein levels was determined. Additionally, the ascorbate level in the blood plasma was analyzed. Before treatment, the ALL patients had profoundly higher levels of the analyzed modified DNA in their urine than the controls. After chemotherapy, we observed a statistically significant decrease in active demethylation products in urine, with a final level similar to the level characteristic of healthy children. The level of 5-hmdC in the DNA of the leukocytes in blood of the patient group was significantly lower than that of the control group. Our data suggest that urinary excretion of epigenetic DNA modification may be a marker of pediatric ALL status and a reliable marker of chemotherapy response.

## Introduction

Acute lymphoblastic leukemia (ALL) is the most common pediatric malignancy. ALL can develop from any lymphoid cell blocked at a particular stage of development and usually originates in a single B- or T-lymphocyte progenitor^1^. Most patients diagnosed with ALL have genetic aberrations that contribute to a higher rate of proliferation and impaired differentiation of lymphoid hematopoietic progenitors^2^.

One reason for the observed genetic alterations may be aberrant DNA methylation that leads to changes in the expression of hematopoietic genes. Moreover, in several studies, it has been observed that the cytosine methylation pattern of leukemic cells differs from that of nonleukemic cells^3–5^.

**Figure 1:**
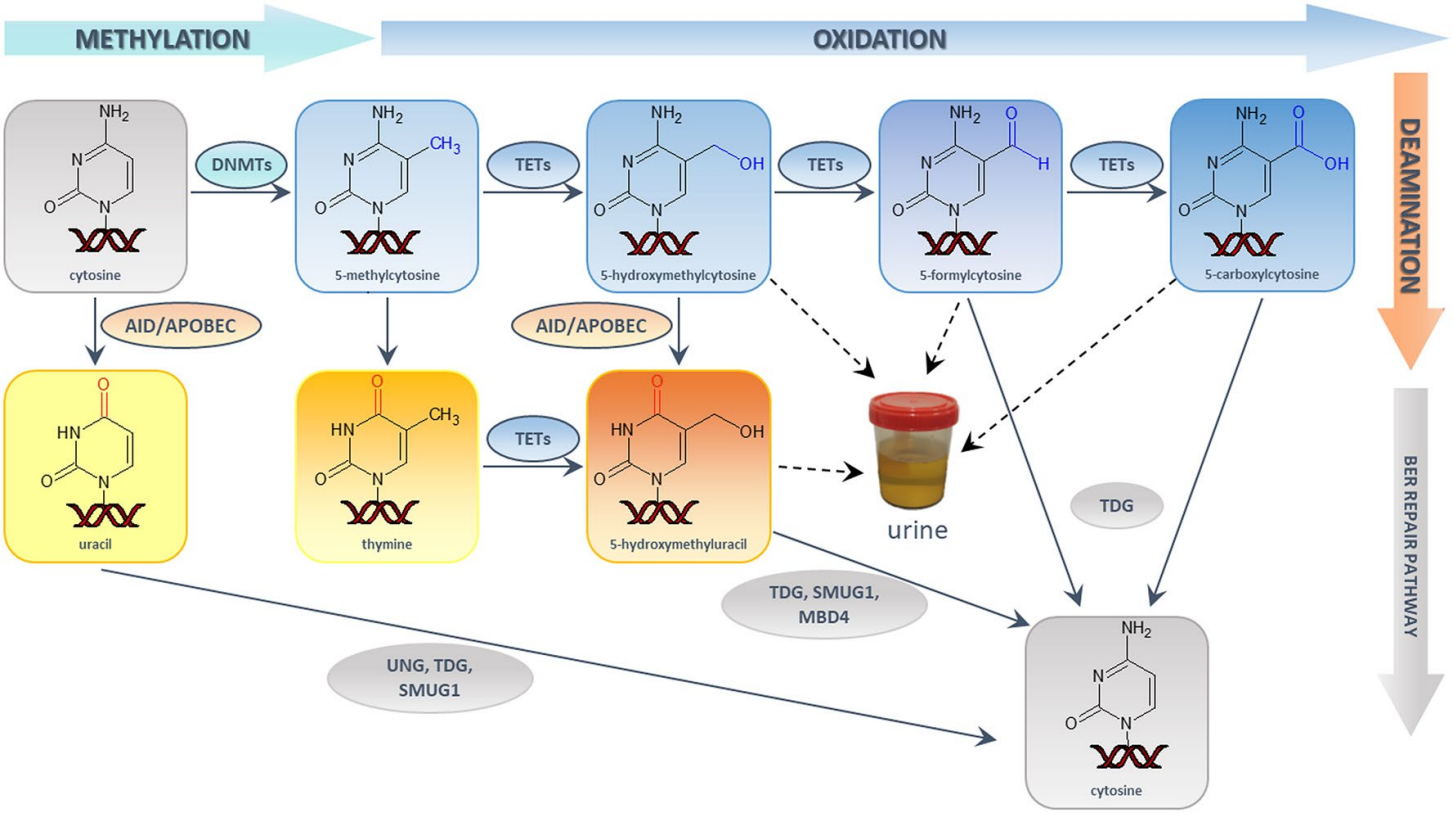
The cytosine methylation and active demethylation pathway. (*DNMT*4: DNA methyltransferase, *TETs: *ten eleven translocation enzymes, *AID:* activation-induced cytosine deaminase, *APOBEC:* apolipoprotein B mRNA editing enzyme, *TDG:* thymine DNA glycosylase, *SMUG1:* single-strand-selective monofunctional uracil-DNA glycosylase 1, *UNG:* uracil-DNA glycosylase, *MBD4:* methyl-CpG-binding domain protein 4, *BER:* base excision repair). Reprinted from Biochimica et Biophysica Acta—Reviews on Cancer, 1869 (1), Olinski R, Gackowski D, Cooke M. Endogenously generated DNA nucleobase modifications source, and significance as possible biomarkers of malignant transformation risk, and role in anticancer therapy, 29–41 (2018), with permission from Elsevier.

DNA methylation has been studied as a stable epigenetic modification for decades. However, despite its stability, DNA methylation may be reversed via an active DNA demethylation process, as described recently (Fig. 1). This demethylation process involves 5-methylcytosine (5-mCyt) enzymatic oxidation (with Ten Eleven Translocation enzymes –TETs) with the subsequent formation of 5-hydroxymethylcytosine (5-hmCyt), which can be further oxidized to 5-formylcytosine (5-fCyt) and 5-carboxylcytosine (5-caCyt)^6,7^. Specific effective enzymatic systems (base excision repair (BER) or nucleotide excision repair (NER)) are needed to remove 5-fCyt and 5-caCyt from DNA and complete the process of active DNA demethylation^8^. Thymine DNA glycosylase (TDG) was demonstrated to be the key BER enzyme involved in the excision of the aforementioned modifications from DNA, i.e., TETs, together with TDG, facilitate active DNA demethylation. 5-hydroxymethyluracil (5-hmUra) may also be generated by TET enzymes and removed via the BER pathway. The eliminated bases (or deoxynucleosides) are released into the bloodstream and eventually also appear in urine^9^.

It is likely that active DNA demethylation is linked with aberrant methylation and may be involved in leukemogenesis. Indeed, hematological malignancies were among the first malignancies in which aberrant demethylation status was discovered, and the TET gene was initially defined as a fusion partner of the mixed lineage leukemia (MLL) gene in acute myeloid leukemia (AML). Importantly, TET inactivation leads to the dramatic deregulation of hematopoiesis, which, in turn, triggers blood malignancies^10^.

The quantification of epigenetic DNA modifications may elucidate the consequences of *TET* expression deficiency. The functional consequences of epigenetic changes may be critical for disease initiation and progression. However, changes in the level of epigenetic DNA modifications may also mirror environmental changes linked with drug delivery.

Acquiring an accurate measurement of 5-formyl-2′-deoxycytidine (5-fdC), 5-carboxy-2′-deoxycytidine(5-cadC) and 5-(hydroxymethyl)-2′-deoxyuridine (5-hmdU) is challenging not only because these modified 2’-deoxynucleosides in mammalian genomes expressed at very low levels but also because the level of 5-(hydroxymethyl)-2′-deoxycytidine (5-hmdC) is 3–4 orders of magnitude higher than the levels of the other forms of modified DNA, which affects the detection and quantitation of the less common metabolites. To address this difficulty, we recently developed a rapid, highly sensitive and specific isotope-dilution automated two-dimensional ultra-performance liquid chromatography with tandem mass spectrometry (2D-UPLC–MS/MS) method that is specifically tailored to simultaneously analyze global levels of 5-mCyt,5-hmCyt, 5-fCyt, 5-caCyt and 5-hmUra (as free bases and 2’-deoxynucleosides) in cellular DNA and urine^11,12^. Importantly, many previous studies have used less reliable semiquantitative immunohistochemical methods to assess the levels of 5-hmC, 5-fC and 5-caC, and the results of these studies have low reliability because of the low levels of modification in genomic DNA^13^.

We thus sought to determine an array of endogenously generated DNA base modifications (5-hmUra, 5-mCyt, 5-hmCyt, 5-fCyt, 5-caCyt). TET enzyme expression at the mRNA and protein levels was also studied.

Chronic inflammation is one of the factors involved in ALL promotion^14^. Since both ALL development and chronic inflammation are associated with oxidative stress in addition to epigenetic DNA modifications, we also determined an established marker of oxidative stress/oxidatively modified DNA - 8-oxo-7,8-dihydro-2′-deoxyguanosine (8-oxodG). Ascorbate (AA) is a potent antioxidant and plays an important role in the active demethylation process as a TET cofactor. Thus, we further analysed the AA concentration in blood samples.

Taking into account the above mentioned events, the aim of the study was to answer the question whether the products of the active DNA demethylation process and oxidative stress may serve as biomarkers for ALL disease status.

## Results

### Levels of epigenetic modifications in urine and DNA

**Figure 2:**
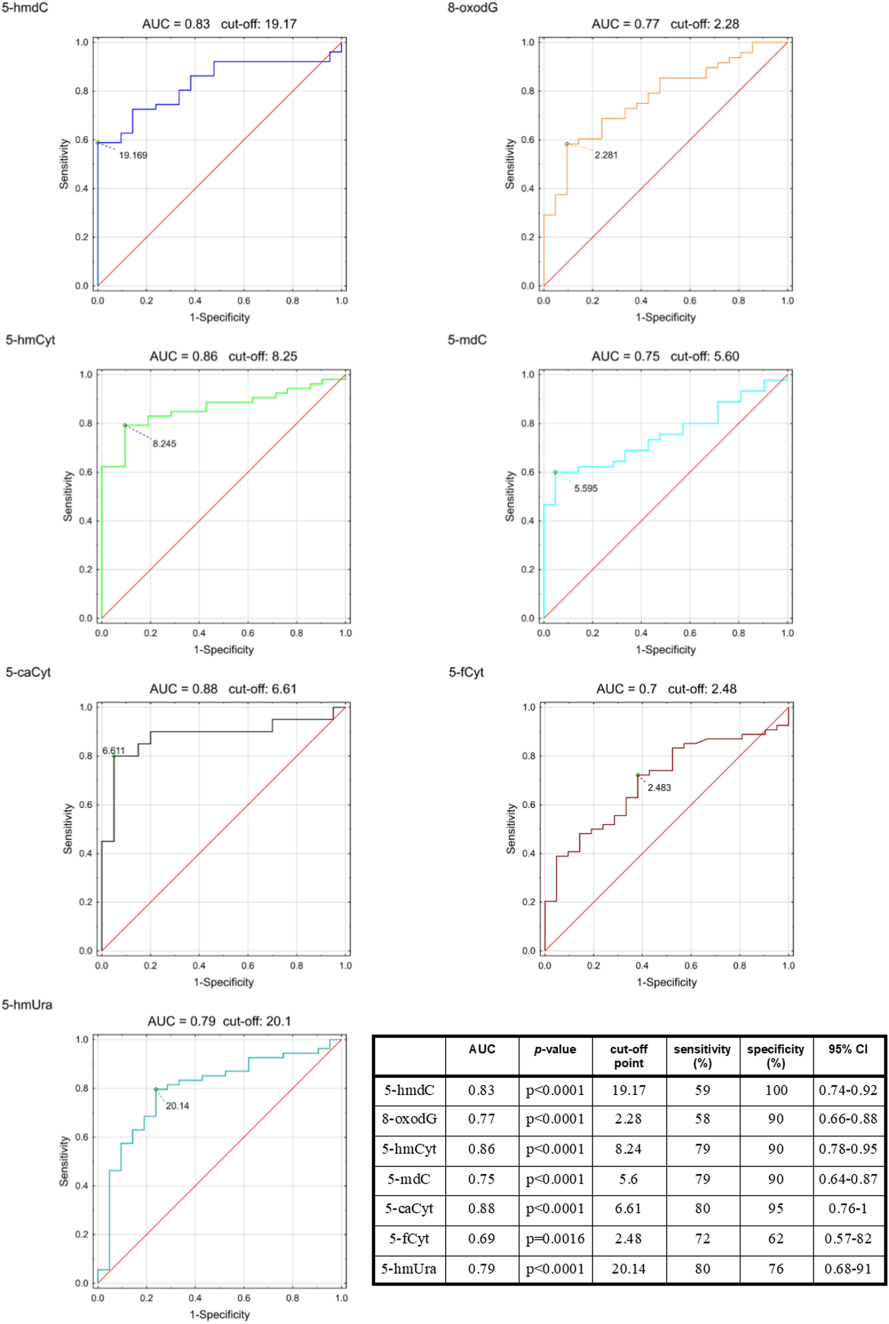
Receiver operating characteristic (ROC) curves for epigenetically modified DNA markers in urine (∗p<0.05). AUC area under the curve.

**Figure 3:**
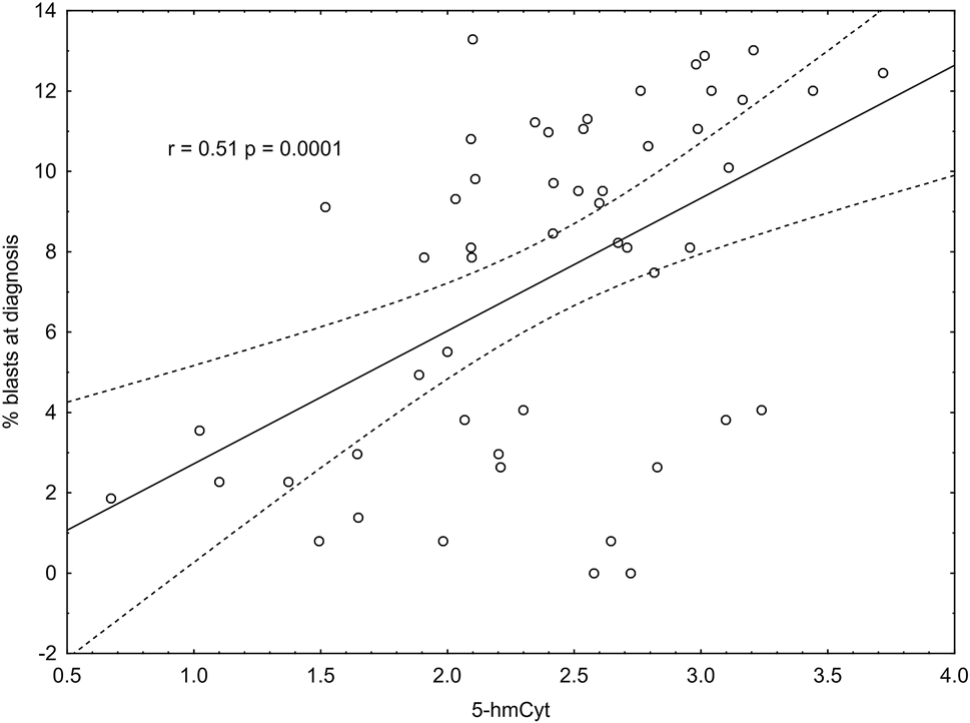
Correlation between 5-hydroxymethylcytosine and % blasts at diagnosis.

**Figure 4:**
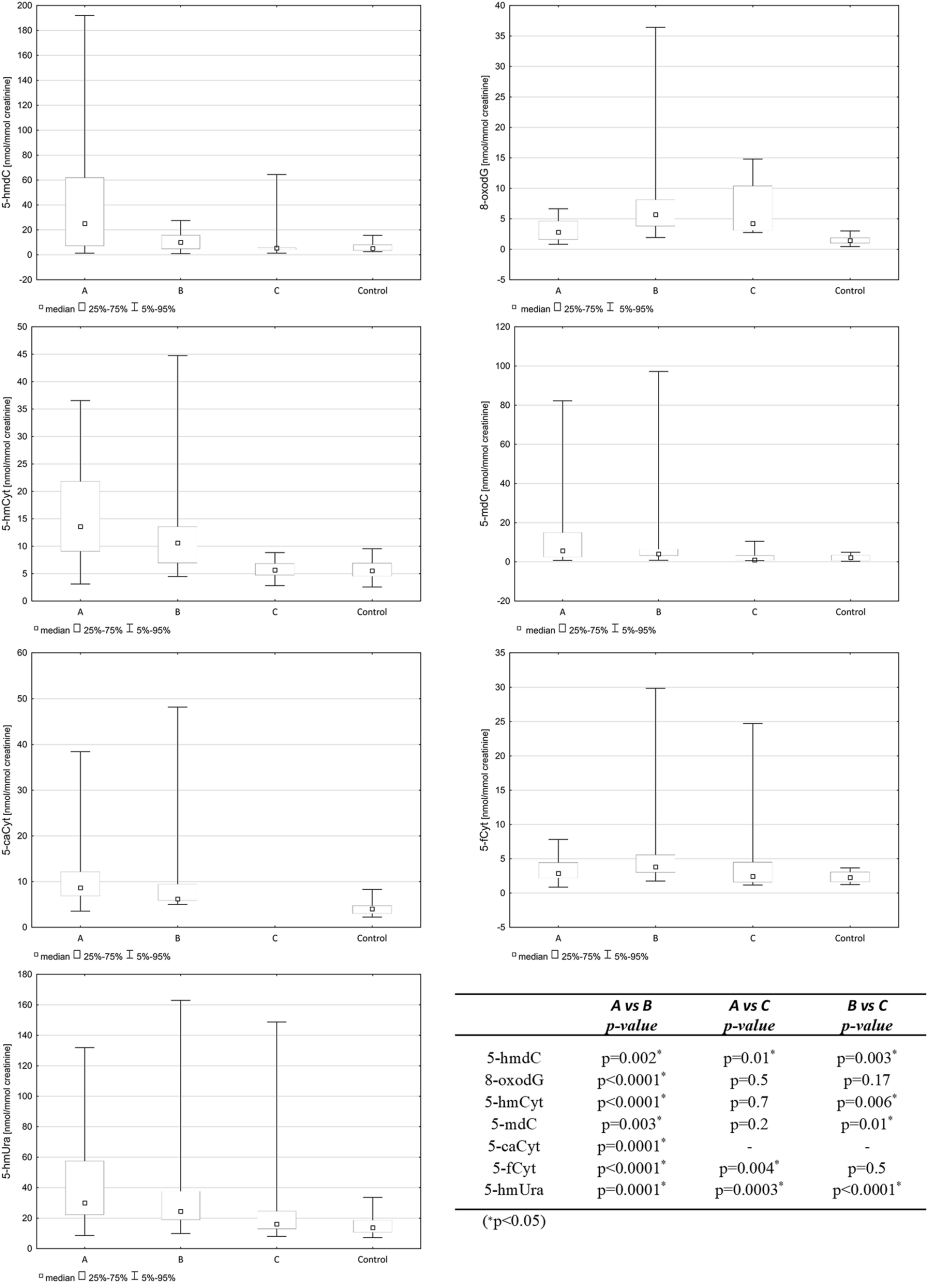
Urinary levels of DNA damage markers and active demethylation products of 5-methylcytosine in healthy controls and ALL patients before therapy (A), 33 days after treatment (B) and six months after treatment (C). The results are presented as median values (∗p<0.05).

Before treatment, the ALL patients had substantially higher levels of analyzed modifications in their urine than the controls, and the differences were significant for all compounds (Table 1). Receiver operating characteristic (ROC) curve analysis showed that the areas under the curve (AUCs) for 5-hmCyt, 5-hmdC and 5-caCyt were higher than 0.8 and statistically significant. The AUCs, the cut-off points optimized for sensitivity and specificity, and the sensitivities and specificities obtained for all urinary metabolites are reported in Fig. 2. A highly significant, good correlation was found between the number of blasts and urinary 5-hmC levels (Fig. 3). After chemotherapy, the patients exhibited a statistically significant decrease in active demethylation products in their urine (5-hmCyt, 5-hmdC, 5-mdCyt, 5-caCyt, 5-hmUra) both 33 days after starting the treatment (point B) and 6 months after the start of therapy (point C). Notably, the levels of analyzed compounds at point C were similar to those observed in the control group (Fig. 4). The level of 5-hmdC in the DNA of leukocytes in the blood of the patient group was significantly lower than that of the control group (median value: 0.065 vs. 0.078, *p* = 0.007, Table 2). A description of the initial diagnosis based on immunophenotypes and genetic aberrations is provided in the Supplementary Materials (Table S1).

**Table 1:** Urinary levels of DNA damage markers and active demethylation products of 5-methylcytosine. The results are presented as median values with interquartile ranges (∗ *p* < 0.05).

	Patients [nmol/mmol creatinine]	Controls [nmol/mmol creatinine]	*p* value
5-hmdC	26.3 (7.6–61.9)	5.1 (3.6–8.0)	0.000001*
8-oxodG	2.5 (1.6–4.0)	1.4 (1.0–1.9)	0.0001*
5-hmCyt	13.6 (8.7–21.8)	5.5 (4.5–6.9)	0.000001*
5-mdC	9.1 (2.2–22.7)	2.2 (0.7–4.3)	0.00003*
5-caCyt	8.7 (6.7–15.1)	4.0 (3.0–4.7)	0.00001*
5-fCyt	3.2 (2.2–4.4)	2.3 (1.6–3.1)	0.01*
5-hmUra	30.4 (22.2–55.2)	13.8 (10.7–18.6)	0.0001*

**Table 2:** The levels of active demethylation products of 5-methylcytosine and 8-oxodG in leukocyte DNA. The results are presented as median values with interquartile ranges (^∗^*p* < 0.05).

	Patients (before treatment)	Controls	*p* value
5-metdC/10e^3^dN	8.7 (8.6–9.0)	8.8 (8.6–9.0)	0.41
5-hmdC/10e^3^dN	0.065 (0.04–0.08)	0.078 (0.07–0.08)	0.007*
5-fdC/10e^6^dN	0.15 (0.1–0.23)	0.15 (0.13–0.22)	0.55
5-cadC/10e^9^dN	nd	nd	–
dU/10e^6^dN	5.5 (3.3–6.3)	4.9 (4.5–5.3)	0.72
5-hmdU/10e^6^dN	0.47 (0.31–0.63)	0.34 (0.23–0.59)	0.28
8-oxodG/10e^6^dN	1.59 (1.2–3.2)	1.38 (1.2–1.6)	0.009*

The ALL patients were divided based on disease subtype (B and T type), and all the analyzed parameters in urine were determined separately by subtype (Table S2 in Supplementary Materials). ETV6/RUNX1 gene fusion generated by t(12;21)(p13;q22) is the most common chromosomal abnormality in children with B-ALL^15–17^. The ETV6/RUNX1 fusion gene was detected in 15 patients (28%). They had substantially higher levels of the analyzed modifications in their urine (except 8-oxodG) than the ETV6/RUNX1-negative ALL patients did, and the differences were significant for 5-hmdC, 5-hmCyt, 5-fCyt and 5-hmUra (Table S3 in Supplementary Materials). Notably, after the completion of chemotherapy, the exceedingly high values dropped to levels observed in the healthy children/control group (see Table S4 in Supplementary Materials). However, in the case of DNA, no statistically significant differences were found. We did not find a correlation between urinary and leukocyte markers.

### Expression of TET and TDG mRNA

The expression of *TET1* mRNA in the ALL patients was significantly higher than that in the controls. The expression of *TET2* mRNA and *TET3* mRNA in the individuals with ALL was significantly lower than that in the controls (*p* = 0.005 and *p* = 0.00004, respectively). A similar statistically significant difference was found for the expression of *TDG* mRNA in the ALL group and the control group (*p* = 0.00001, Table 3).

**Table 3:** Expression of *TET1*, *TET2*, *TET3* and *TDG* mRNA in the healthy controls and ALL patients (^**∗**^ *p* < 0.05).

Gene mRNA	ALL [mRNA expression ratio]	Control [mRNA expression ratio]	ALL vs. control *p* value
*TET1*	0.002 (0.001–0.012)	0.001 (0.0007–0.0015)	0.013*
*TET2*	0.69 (0.52–0.97)	1.08 (0.92–1.61)	0.005*
*TET3*	0.19 (0.059–0.35)	0.39 (0.34–0.65)	0.00004*
*TDG*	0.47 (0.32–0.78)	0.23 (0.2–0.3)	0.00001*

### Protein expression analysis

The protein expression of TET1 in the ALL group was significantly lower than that in the control group (*p* = 0.007). In the case of the TET2, TET3 and TDG proteins, we found no statistically significant differences (Table 4). The protein expression of TDG significantly decrease after treatment (median value 1.728 vs 1.428 respectively, *p* = 0.037, Fig. S1 in Supplementary Materials).

**Table 4:** Expression of TET1, TET2 and TET3 protein in the lymphocytes of healthy controls and ALL patients (^**∗**^*p* < 0.05).

Protein	ALL [protein level]	Control [protein level]	ALL vs. control *p* value
TET1	1.19 (1.04–1.62)	1.6 (1.4–1.92)	0.007*
TET2	1.3 (1.08–1.91)	1.48 (1.3–1.71)	0.52
TET3	2.45 (1.7–3.94)	3.25 (2.36–3.61)	0.35
TDG	1.8 (1.12–2.63)	1.6 (1.42–1.86)	0.083

Interestingly, significant correlations between TET (“producer”) and TDG (“eraser”) proteins were found (Fig. S2–S5 in supplementary materials). Moderate positive correlations were observed between active DNA demethylation products and TET proteins in selected cell populations (Fig. S6 in supplementary materials).

### Differences in the plasma concentrations of ascorbate and 8-oxodG levels in blood and urine

Significantly elevated levels of 8-oxodG were noted in the urine of patients after chemotherapy (2.8 nmol/mmol creatinine at point A and 5.7 and 4.2 at points B and C). In the case of 8-oxodG in cellular DNA, the ALL patients showed significantly higher levels than the controls (1.59 vs. 1.38, respectively, *p* = 0.009, Table 2).

**Figure 5:**
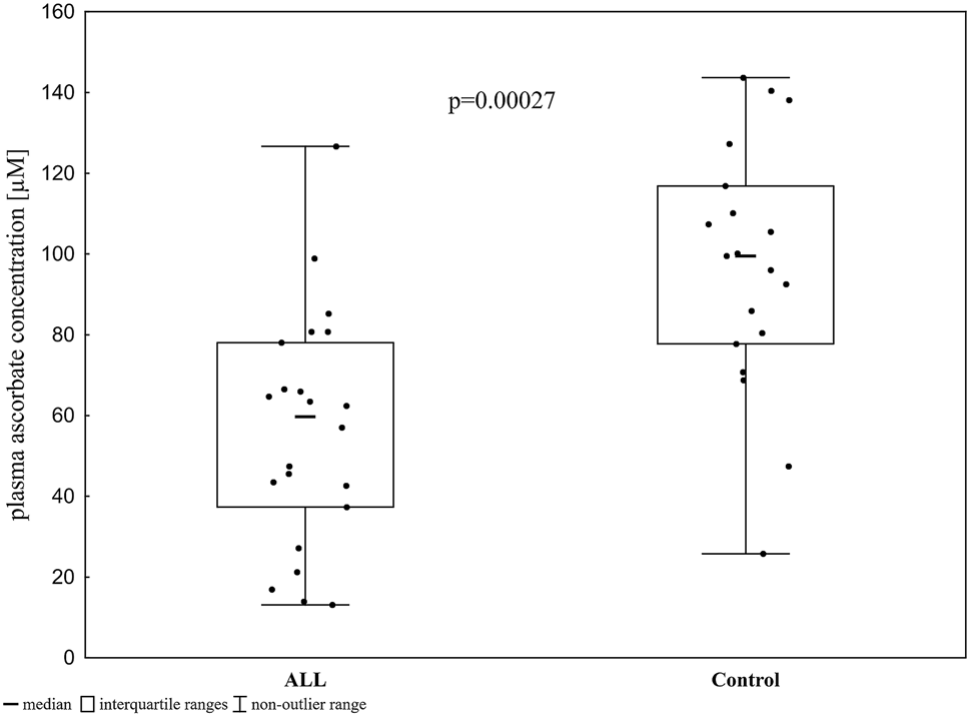
Ascorbic acid in plasma.

Plasma concentrations of ascorbate in the ALL patients were lower than those in the controls (median value 62.4 vs. 97.8 µM, respectively, *p* = 0.00027, Fig. 5), which suggests an antioxidative role of AA.

## Discussion

Many studies have implicated aberrant epigenetic regulation in the pathogenesis and treatment outcome of ALL. The active DNA demethylation process may be linked to aberrant methylation and may be involved in leukemogenesis.

We found a significant decrease in a key product of active demethylation, 5-(hydroxymethyl)-2′-deoxycytidine (5-hmdC), in the leukocyte DNA of ALL patients compared with that of healthy subjects. A similar decrease in this epigenetic modification has been found in many types of human malignancies^13,18–20^. One of many possible reasons for the loss of 5-hmCyt is the decreased expression of *TET* mRNA^18,21^. Consistent with this supposition, we found decreases in *TET3* and *TET2* but increases in *TET1* mRNA expression in the leukocytes of ALL patients. However, at the protein level, the only significant difference inexpression between the examined tissues was found for *TET1*, which was significantly more abundant in the control group than in the ALL patients. A likely reason for the changes in *TET* mRNA expression is methylation of CpG sites in the promoter region, which was found to correlate with gene expression changes in primary ALL cells^15^. Several factors may affect TET activity and thereby either enhance or attenuate enzyme effects. For instance, posttranslational context-dependent acetylation and deacetylation of TET2 lysine residues may enhance or reduce TET2 stability and activity^22,23^. Moreover, interactions with multiple binding partners may recruit TETs to or exclude TETs from specific genomic sites^24,25^. However, key questions concerning the relationship among individual TETs, the relation between *TET* mRNA and TET proteins and the connection between the expression and activity of the enzymes remain largely unanswered.

Another factor that influences the activity of TET proteins is vitamin C. Previous studies demonstrated that ascorbate may enhance the generation of 5-hmCyt in cultured cells and under in vivo conditions^26–28^. Ascorbic acid (AA) enhances the activity of TETs, likely at the active site, by reducing Fe^3^^+^ to Fe^2+^, which enables the donation of the electron needed to split an O–O into hydroxyl groups. Interestingly, the ALL patients analysed in our study had significantly lower serum AA concentrations (Fig. 5). Thus, a shortage of vitamin C may be another factor critical to the observed decrease in 5-hmC levels in the leukocyte DNA of the patient group (in addition to decreased TET expression).

In the ALL group, patients with ETV6/RUNX1 gene fusion (also known as TEL/AML1) had higher levels of analyzed modifications in urine, and for 5-hmdC, 5-hmCyt, 5-fCyt and 5-hmUra, the differences were significant (Table S3 in Supplementary Materials). Interestingly, a bias towards hypermethylation has been found in ETV6/RUNX1-positive pediatric ALL patients^4,29^. However, in other studies, both higher and lower methylation statuses in ALL patients with fusion were found^15^. In the case of hyperdiploidy, no differences between the subtypes were found.

In patients treated with chemotherapy (methotrexate or epirubicin), the 8-oxodG levels in leukocyte DNA and urine were increased almost two fold compared to those before treatment. One component of the therapy drug cocktail is the anthracycline derivative epirubicin. The cytotoxicity of these drugs has been attributed to the inhibition of topoisomerase II and the intracellular production of free radicals. In our previous study, as in this study, significant increases in the amount of oxidatively modified DNA lesions, including 8-oxodG, were found in the lymphocytes of cancer patients treated with epirubicin^30^.

The excretion of epigenetic DNA modification into urine is equal to the rate of active demethylation of DNA. This in turn implies that the increase in the urinary excretion of modified DNA observed in our study should be determined by disease development. Since we observed only subtle differences in the modification levels of cellular DNA (in 5-hmdC only, with no changes in the other modifications), the increase may solely represent alterations in the rate of repair.

The urinary excretion rates of the majority of the DNA epigenetic modifications were significantly higher in the patient group than in the healthy group (Table 1). Recently, we and others found significant changes in the epigenetic modifications of DNA in the urine only for 5-hmdC inpatients with solid tumours and lymphoma patients^12,31^. Therefore, the results presented in this work may indicate the essential effects of whole spectrum of epigenetic changes in DNA on the malignant transformation observed in pediatric ALL.

Importantly, a profound decrease in these modifications to the levels observed in the urine of the healthy subjects was observed after the patients completed chemotherapy (Fig. 4). The likely reason for this decrease is the massive death of cells with a high proliferative rate, namely, cancer cells. In addition, cancer cells have a higher turnover of epigenetic markers. Indeed, a highly significant, good correlation was found between the number of blasts and urinary 5-hmC levels (Fig. 3). In the majority of the ALL patients, the leukemic blast fraction comprised 30–91% of all WBC cells, while after chemotherapy, the number of leukemic blasts was undetectable in the great majority of patients (Table S1). Therefore, the abovementioned decrease may be linked with the patients’ recovery. Moreover, one of the components of ALL treatment, methotrexate (MTX), acts by inhibiting dihydrofolate reductase, which is critical for the reduction in methyltetrahydrofolate and for methylation during DNA replication, which in turn directly reduces cell proliferation^32^. Furthermore, MTX is directly involved in the inhibition of DNA methylation in pediatric leukemia patients^33^.

Normal cell development requires the tight control of the DNA methylation pattern, which is involved in the regulation of gene expression. Cytosine methylation, usually in CpG dinucleotides, is a key epigenetic modification that exerts a profound impact on gene repression, cellular identity and the maintenance of genome stability^34^. The opposite reaction, namely, active DNA demethylation, is equally important to the DNA methylation pattern and can result in the activation of previously silenced genes. This process is directly linked to DNA repair and the removal of epigenetic modifications such as 5-fCyt and 5-caCyt (also 5-hmUra), which are replaced by unmodified cytosine. The removed bases (or deoxynucleosides) are released into the bloodstream and eventually appear in the urine^35^.Therefore, the most plausible source of the modifications excreted in the analyzed urine is the DNA repair process. Both 5-fCyt and 5-caCyt may inhibit DNA replication, which may result in genome instability^36,37^. Therefore, effective enzymatic systems are involved in the removal of modifications from DNA. Thus, TDG has robust excision activity towards 5-fCyt and 5-caCyt^38–40^. The activity of the abovementioned enzymes may contribute to the presence of modified bases in urine. Importantly, in the present study, the expression of *TDG* mRNA was sharply increased in the ALL patients compared with controls (Table 3).

Another source of the epigenetic markers excreted in urine that were analyzed in our study may be the processive demethylation pathway, as proposed by Franchini et al.^41^. That is, 5-fCyt, 5-caCyt and 5-hmUra initiate processive DNA demethylation, which is primarily a single initiating event (such as a certain mismatch) that may trigger the processive demethylation of numerous 5-mCyts (and perhaps 5-hmCyts) on the same locus via the long-patch BER, DNA mismatch repair (MMR) or nucleotide excision repair (NER) pathways. Recent studies have demonstrated that 5-hmUra may trigger the removal of distant epigenetic modifications (5-mCyt and 5-hmCyt) through MMR- and long-patch BER-dependent pathways^42^. These findings may explain the presence of 5-hmCyt and 5-mCyt deoxynucleosides in urine.

Interestingly, the urinary excretion rates found in this study for the control group/healthy children were similar to those we obtained for healthy adults, as described in our recently published study^12^. These findings seem to reflect basic processes that govern metabolic pathways critical for maintaining certain patterns of epigenetic DNA markers.

Several recently published studies have demonstrated that certain epigenetic alterations are essential for leukemic transformation^15^. The results of the aforementioned studies demonstrated the hypermethylation of GC islands in all subtypes of ALL. However, in these ALL cells, genome-wide hypomethylation was also frequently detected. Moreover, the ALL genomes were characterized by few somatic mutations, while the depletion of epigenetic modifications was simultaneously exacerbated in the majority of the pediatric ALL patients^15^. It is likely that profound changes in the urinary excretion rates of epigenetically modified DNA in ALL patients directly reflect the abovementioned dynamic changes in methylation signatures during disease development.

A large body of evidence suggests that the level of 5-hmCyt in the cellular DNA of human malignancies is substantially reduced^13,18–20^. Moreover, recently published data suggest that the decrease in the 5-hmCyt level in DNA may serve as a biomarker of early malignant transformation and can be used as a prognostic factor for patients with malignancies^20,43^. Consequently, the urinary modifications described herein may be used as potential risk and response markers. Of note, ROC curve analysis confirmed high sensitivity and specificity for 5-hmCyt, 5-hmdC and 5-caCyt (Fig. 2). Given the difficulties of obtaining specimens/tissues, such as bone morrow, especially from pediatric patients, the determination of epigenetic DNA modifications in human urine may serve as an attractive noninvasive diagnostic option. Furthermore, the noninvasiveness of the test constitutes a strong argument for its application to large-scale basic research and clinical studies dealing with the role of active demethylation in carcinogenesis.

Our results suggest that the urinary excretion of epigenetically modified DNA may be a marker of ALL status and a reliable marker of chemotherapy response. Of note, the differences in these markers between ALL patients and the control group are so clear that they may be detected by commercially available immunochemical tests.

## Materials and methods

The subjects of the study were children (n = 55) with newly diagnosed acute lymphoblastic leukemia recruited from a hospital setting (Department of Pediatric Hematology and Oncology, Collegium Medicum in Bydgoszcz, Nicolaus Copernicus University Torun, Jurasz University Hospital, Bydgoszcz, Poland). The patients were treated according the ALL-IC-2009 or AIEOP-BFM ALL-2017 protocols. Peripheral blood and urine samples were collected before treatment began (point A), 33 days after the initiation of treatment (point B) and 6 months after the initiation of treatment (point C – urine only). Samples obtained from healthy donors were used as controls (n = 21) after informed consent was obtained from their legal guardians. The clinical details of the patients are shown in Table 5. No active cytomegalovirus or Epstein–Barr virus infections were found in the studied groups. The standard panel of antibodies used to determine the immunophenotypic characterization included CD45, CD19, CD20, CD10, CD38 and CD58 for precursor-B-lineage and TdT, CD45, CD7, CD3, CD4, CD8, CD99, and cytoplasmic CD3 for T-lineage ALL^44^. All clinical investigations were conducted according to the principles of the Declaration of Helsinki. The study protocol was approved by the local Bioethics Committee at Collegium Medicum in Bydgoszcz, Nicolaus Copernicus University (KB 404/2016), and the guardians of all the patients provided written informed consent.

**Table 5:** Baseline characteristics of the study groups.

	Patients, n = 55	Controls, n = 21
Age (years) Median (range)	5 (1–18)	8 (2–17)
Sex Male/female (N)	33/22	13/8
Platelets [/µl] Median (range)	67,000 (125–457,000)	
WBC at diagnosis [/µl] Median (range)	4840 (33–5,170,000)	
% Peripheral blasts at diagnosis Median (range)	35 (0–95)	
% Bone morrow (BM) blasts at diagnosis Median (range)	91 (29–98)	
Immunophenotype, n (%) T‐ALL B‐ALL	6 (11) 49 (89)	

### Determination of epigenetic modifications and 8-oxodG levels in urine

Two-dimensional ultra-performance liquid chromatography with tandem mass spectrometry (2D UPLC–MS/MS) was used for the epigenetic modification analysis of urine samples (with the exception of 5-hmUra). The 2D-UPLC − MS/MS system consists of a gradient pump and autosampler for one-dimensional chromatography, and a gradient pump and tandem quadrupole mass spectrometer with a UNISPRAY ion source was used for two-dimensional chromatography. Due to the low sensitivity of the method used, the 5-hmUra level was determined by high-performance liquid chromatography for prepurification followed by gas chromatography with isotope dilution mass spectrometric detection (LC/GC–MS), as previously described^45^.

### Isolation of DNA and the determination of epigenetic modifications and 8-oxodG in DNA isolates

Leukocytes were isolated from heparinized blood samples with Histopaque 1119 (Sigma) solution according to the manufacturer’s instructions and stored at − 80 °C until analysis. The analyses were performed using a method previously described by Gackowski et al.^11^.

### Determination of ascorbate levels in blood plasma by UPLC‑UV

The analyses were performed using a method previously described by Starczak et al., with some modifications^46^.

Details of the methods and additional methods (gene and protein expression analysis) are provided in the Supplementary Materials.

### Statistical analysis

The results are presented as median values, interquartile ranges and nonoutlier ranges. Statistical analyses were carried out with Statistica 13.1 PL software [Dell, Inc. (2016). Dell Statistica, version 13]. Normal distribution of the study variables was verified with the Kolmogorov–Smirnov test with Lilliefors correction and based on visual inspection of plotted histograms. Variables with a normal distribution were analyzed as raw data, while variables with nonnormal distributions were subjected to Box–Cox transformation prior to statistical analyses based on parametric tests. The univariate unpaired Student's t test (two-tailed) was used to compare groups, and the paired Student's t test was used for pre- and posttreatment analysis. Receiver operating characteristic (ROC) curve analysis was used to assess the sensitivity, specificity, and area under the curve (AUC) of epigenetic DNA modifications in urine. The results were considered statistically significant at *p* values less than 0.05.

## Supplementary Information

Supplementary Information.

## Data Availability

The datasets obtained in the current study are available from the corresponding author on reasonable request.

## Abbreviations

ALL: Acute lymphoblastic leukemia
TET: Ten-eleven translocation
5-mdC: 5-Methyl-2′-deoxycytidine
5-mCyt: 5-Methylcytosine
5-hmCyt: 5-Hydroxymethylcytosine
5-fCyt: 5-Formylcytosine
5-caCyt: 5-Carboxycytosine
5-hmUra: 5-Hydroxymethyluracil
2D-UPLC-MS/MS: Two-dimensional ultra-performance liquid chromatography with tandem mass spectrometry
8-oxodG: 8-Oxo-7,8-dihydro-2′-deoxyguanosine
5-hmdC: 5-(Hydroxymethyl)-2′-deoxycytidine
5-fdC: 5-Formyl-2′-deoxycytidine
5-cadC: 5-Carboxy-2′-deoxycytidine
5-hmdU: 5-(Hydroxymethyl)-2′-deoxyuridine
MLL: Mixed lineage leukemia
AML: Acute myeloid leukemia
LC/GC–MS: Liquid chromatography followed by gas chromatography with isotope dilution mass spectrometric detection
UPLC‑UV: Ultra-performance liquid chromatography coupled with UV detection
BER: Base excision repair
MMR: DNA mismatch repair
NER: Nucleotide excision repair
RT-qPCR: Quantitative real-time PCR
