# Supplementary material for: The urinary excretion of epigenetically modified DNA as a marker of pediatric ALL status and chemotherapy response
Source: Sci Rep. 2021 Nov 1;11:21345. doi: 10.1038/s41598-021-00880-9 (PMC8560782; doi:10.1038/s41598-021-00880-9)
Supplement: Supplementary file 1 — Supplementary Information. [file 41598_2021_880_MOESM1_ESM.pdf]

The urinary excretion of epigenetically modified DNA as a marker of pediatric ALL status and chemotherapy response.

Rafal Rozalski[1*], Daniel Gackowski[1], Aleksandra Skalska-Bugała [1], Marta Starczak[1], Agnieszka Siomek-Gorecka[1], Ewelina Zarakowska[1], Martyna Modrzejewska[1], Tomasz Dziaman[1], Anna Szpila[1], Kinga Linowiecka[1,2], Jolanta Guz[1], Justyna Szpotan[1,2], Maciej Gawronski[1], Anna Labejszo[1], Lidia Gackowska[3], Marek Foksinski[1], Elwira Olinska[4,], Aleksandra Wasilow[1], Andrzej Koltan[5] and Jan Styczynski[5] & Ryszard Olinski[1*]

Supplementary Materials

Page No.

Table S1. Immunophenotypes and genetic aberrations in the ALL group 2

Table S2. Urinary levels of DNA damage markers and active demethylation products of 5-methylcytosine 3

Table S3. Urinary levels of DNA damage markers and active demethylation products of 5-methylcytosine in ETV6/RUNX1 4

Table S4. Urinary levels of DNA damage markers and active demethylation products of 5-methylcytosine in ETV6/RUNX1-negative ALL (I) and ETV6/RUNX1-positive ALL (II) group before therapy (A), 30 days after treatment (B) and six months after treatment (C) 5

Figure S1. Expression of TDG protein in the leukocytes before starting the treatment and 33 days after the initiation of treatment 6

Figure S2. Correlations between TDG and TET proteins in atypical cells 7

Figure S3. Correlations between TDG and TET proteins in granulocytes 8

Figure S4. Correlations between TDG and TET proteins in monocytes 9

Figure S5. Correlations between TDG and TET proteins in lymphocytes. Individual data points represent Box-Cox transformed values 10

Figure S6. Correlations between the active DNA demethylation products and TET proteins in selected cell populations 11

Supplementary Methods 12-14

Table S5. Primers and short hydrolysis probes used for the target gene mRNA expression analysis........ 13

Table S6. Panel of surface labelling markers 14

Table S7. Panel of intracellular labelling........ 15

Supplementary References 16

Table S8. Transition patterns and specific detector settings for all compounds analyzed by 2D UPLC–MS/MS in urine 17

Table S1. Immunophenotypes and genetic aberrations in the ALL group.

	Immunophenotype			Immunophenotype (detailed)	karyotype t(4;11)	karyotype t(9;22)	hypodiploidy	hyperdiploidy	BCR/ABL	KMT2A-AFF1 (MLL/AF4)	ETV6-RUNX1 (TEL/AML1)	% blasts at diagnosis	day 33 % blasts
1	ALL	T	Mature T	CD3cyt+CD5+CD7+CD3-CD4-CD8-CD99+CD2-	0	0	0	0	0	0	0	35	0
2	ALL	B	common	CD10+CD19+CD20-CD45-CD34+TdT+	0	0	0	1	0	0	0	48	0.4
3	ALL	B	common	CD10+CD19+CD20+/-CD45+CD13+	0	0	0	0	0	0	0	0	0.2
4	ALL	B	common	CD10+CD19+CD20-CD45-CD34-	0	0	0	1	0	0	0	6	0
5	ALL	B	common	CD10+CD19+CD20-CD45-CD34-CD38+HLA-DR+	0	0	0	0	0	0	1	10	1.4
6	ALL	B	common	CD10+CD19+CD20-CD45-CD34+TdT+	0	0	0	1	0	0	0	4	0
7	ALL	B	common	CD10+CD19+CD20-CD45-CD34-CD38+HLA-DR-	0	0	0	0	0	0	1	77	0
8	ALL	B	common	CD10+CD19+CD20-CD45-CD34+TdT-	0	0	0	0	0	0	0	67	0
9	ALL	B	Mature B	CD10+CD19+CD20+CD45+CD34-	0	0	0	0	0	0	0	33	0
10	ALL	B	Pro-B	CD19+CD10-CD20-CD45-CD34-TdT-	0	0	0	0	0	0	0	60	0.4
11	ALL	B	common	CD10+CD19+CD20-CD45-CD34-/+TdT+	0	0	0	0	0	0	0	0	0
12	ALL	B	common	CD10+CD19+CD20-CD45-CD34+CD22-IgM-	0	0	0	1	0	0	0	9	0.4
13	ALL	B	common	CD10+CD19+CD20+/-CD45+CD34-	0	0	0	0	0	0	0	1	0
14	ALL	B	common	CD10+CD19+CD20-CD45-CD34+IgM-	0	0	0	0	0	0	0	38	0.3
15	ALL	B	common	CD10+CD19+CD20-CD45-CD34+IgM-	0	0	0	1	0	0	0	0	0
16	ALL	B	common	CD10+CD19+CD20-CD45-CD34-CD45-TdT+IgM-	0	0	0	0	0	0	1	91	0.6
17	ALL	B	common	CD10+CD19+CD20-CD45-CD34-CD22-IgM-	0	0	0	1	0	0	0	45	0
18	ALL	B	common	CD10+CD19+CD20-CD45-CD34-CD22-IgM-TdT-	0	0	0	0	0	0	1	15	0
19	ALL	B	common	CD10+CD19+CD20+CD45-CD34-TdT+	0	0	1	0	0	0	0	9	0
20	ALL	B	common	CD10+CD19+CD20+CD45-CD34-CD22-IgM-	0	0	1	0	0	0	0	51	0
21	ALL	B	common	CD10+CD19+CD20-CD45+CD34-	0	0	0	0	0	0	0	1	0
22	ALL	B	common	CD10+CD19+CD20-CD34+CD38+HLA-DR+CD45-IgM-	0	0	0	0	0	0	0	65	0.4
23	ALL	T	Mature T	CD3cyt+CD3-CD4-CD8-CD2-CD7+CD34-CD45-CD5-CD99+	0	0	0	0	0	0	0	86	0
24	ALL	B	common	CD10+CD19+CD20-CD45+CD34+CD13+	0	0	0	0	0	0	0	3	0
25	ALL	B	Pro-B	CD19+CD10-CD20-CD34-CD38+HLA-DR+CD45+IgM-	1	0	0	0	0	1	0	83	0
26	ALL	B	common	CD10+CD19+CD20-CD34+CD45-	0	0	0	1	0	0	0	50	0.2
27	ALL	B	common	CD10+CD19+CD20-CD34+CD45-CD22-IgM-TdT+	0	0	0	0	0	0	0	8	0
28	ALL	B	common	CD10+CD19+CD20+CD22-IgM-CD34-CD45-CD38+HLA-DR+	0	0	0	1	0	0	0	14	0.2
29	ALL	B	Pro-B	CD10-CD19+CD20-CD34+CD38+HLA-DR+CD45+TdT-	1	0	0	0	0	0	1	62	0.4
30	ALL	B	common	CD10+CD19+CD20-CD34+TdT+CD45-HLA-DR+	0	0	0	0	0	0	1	95	0
31	ALL	B	common	CD10+CD19+CD20-CD34+CD45-HLA-DR+CD38-TdT+	0	0	0	0	0	0	1	65	0.8
32	ALL	B	common	CD10+CD19+CD20-CD45-CD34+TdT+IgM-	0	0	0	0	0	0	0	36	0
33	ALL	B	common	CD10+CD19+CD20-CD34+TdT+CD45-CD38+HLA-DR+CD22-IgM-	0	0	0	1	0	0	0	64	0
34	ALL	T	Mature T	CD7+CD2-/+CD3cyt-CD3-CD4-CD8-CD5-TdT	0	0	0	0	0	0	0	44	5.2
35	ALL	B	common	CD10+CD19+CD20-CD22-CD34+CD45-CD38+HLA-DR+TdT+	0	0	0	0	0	0	0	1	0.03
36	ALL	B	common	CD10+CD19+CD20-CD79a+CD22-CD34+CD45-TdT+IgM-CD38+HLA-DR+	0	0	0	0	0	0	1	77	0
37	ALL	B	common	CD10+CD19+CD20-CD22-IgM-CD34+TdT+CD45-CD38+HLA-DR+	0	0	0	0	0	0	1	68	0
38	ALL	T	Mature T	CD2+CD5+CD3-CD3 intra+CD4-CD8+CD7+CD1a-CD34-CD45+	0	0	0	0	0	0	0	77	9.8
39	ALL	B	common	CD10+CD19+CD20+CD22+CD34-IgM- CD45+HLA-DR+ TdT+	0	0	0	1	0	0	0	2	1.2
40	ALL	B	common	CD10+CD19+CD20+CD22+CD79alpha+CD34+TDT+CD45-	0	0	0	0	0	0	0	54	0
41	ALL	B	common	CD10+CD19+CD20-/+CD79alpha+CD22+CD34+CD45-IgM-	0	0	0	0	0	0	0	5	0
42	ALL	B	common	CD10+CD19+CD20+CD22+IgM-CD34+TdT-CD45-	0	0	0	1	0	0	1	17	0
43	ALL	B	common	CD10+CD19+CD20-CD34+TdT+CD22-IgM-CD79alfa-	0	0	0	0	0	0	0	48	0.9
44	ALL	B	common	CD10+CD19+CD20-CD34+CD79alpha+CD22+IgM-TdT-	0	0	0	0	0	0	0	4	0.8
45	ALL	B	common	CD10+CD19+CD20-CD79alpha+IgM-CD34+TdT+	0	0	0	0	0	0	0	35	0
46	ALL	B	common	CD10+CD19+CD20-CD34-CD79a+IgM-TdT-CD45-	0	0	0	0	0	0	0	35	0
47	ALL	T	Mature T	CD3cyt+CD2+CD3pow-CD4-CD8-CD5-CD7-CD1a-CD99+CD34+TdT-	0	0	0	0	0	0	0	74	0
48	ALL	B	common	CD10+CD19+CD20-CD79a+CD22+CD34-TdT-	0	0	0	0	0	0	1	89	0
49	ALL	T	Mature T	CD3cyt+CD3+CD2--CD7+CD5+CD4-CD3-CD99+CD34+TdT-	0	0	0	0	0	1	0	33	0
50	ALL	B	common	CD10+CD19+CD20-CD22+CD79a	0	0	0	0	0	0	0	10	0
51	ALL	B	common	CD10+CD19+CD20-CD22+CD79a+CD34+IgM - CD13+ co-expression	0	0	0	0	0	0	1	46	0.4
52	ALL	B	common	CD10+CD19+CD20-CD22+CD79a+CD34+IgM - CD13+ co-expression	0	0	0	0	0	0	1	10	0.8
53	ALL	B	common	CD10+CD19+CD20-CD79a+CD22+CD34+IgM-	0	0	0	0	0	0	1	5	0.2
54	ALL	B	common	CD10+CD19+CD20-CD22+CD79a+IgM-CD34-	0	0	0	0	0	0	1	30	0
55	ALL	B	Pro-B	CD19+CD10-CD20-CD22+IgM-CD79a+CD34+ - CD15+ co-expression	0	0	0	1	0	0	0	6	0

Table S2. Urinary levels of DNA damage markers and active demethylation products of 5-methylcytosine (* $p<0.05$).

[nmol/mmol creatinine]	ALL (N=55)	B-cell ALL (N=49)	T-cell ALL (N=6)	Controls (N=21)	ALL vs Control P value	B-cell ALL vs Control P value	T-cell ALL vs Control P value	B-cell vs T-cell ALL P value
5-hmdC	26.3 (7.6-61.9)	27.6 (7.6-66.3)	11.2 (8.5-26.5)	5.1 (3.6-8.0)	0.000001*	0.0000001*	0.03*	0.18
8-oxodG	2.5 (1.6-4.0)	2.6 (1.9-4.0)	1.4 (1.2-1.6)	1.4 (1.0-1.9)	0.0001*	0.00004*	0.57	0.14
5-hmCyt	13.6 (8.7-21.8)	13.6 (8.4-21.2)	23.1 (9.3-26.6)	5.5 (4.5-6.9)	0.000001*	0.00000002*	0.009*	0.5
5-mdC	9.1 (2.2-22.7)	9.2 (1.7-27.4)	5.6 (3.1-12.3)	2.2 (0.7-4.3)	0.00003*	0.00007*	0.04*	0.8
5-caCyt	8.7 (6.7-15.1)	11.5 (6.9-15.7)	7.2 (2.2-8.7)	4.0 (3.0-4.7)	0.00001*	0.000001*	0.7	0.3
5-fCyt	3.2 (2.2-4.4)	3.3 (2.4-4.5)	2.4 (2.2-3.7)	2.3 (1.6-3.1)	0.01*	0.003*	0.3	0.2
5-hmUra	30.4 (22.2-55.2)	33.4 (24.2-58.6)	21.4 (14.3-23.6)	13.8 (10.7-18.6)	0.0001*	0.00002*	0.5	0.07

The results are presented as median values, and interquartile ranges.

Table S3. Urinary levels of active demethylation products of 5-methylcytosine in ETV6/RUNX1-negative ALL (A) and ETV6/RUNX1-positive ALL (B) group (∗ $p<0.05$). The results are presented as median values and interquartile ranges.

	A [nmol/mmol creatinine]	B [nmol/mmol creatinine]	P value
5-hmdC	13.7 (6.0-41.4)	56.9 (23.9-187.4)	0.0008*
8-oxodG	2.5 (1.6-4.0)	2.4 (1.9-3.9)	0.9
5-hmCyt	13.6 (7.4-19.5)	18.9 (9.4-33.3)	0.04*
5-mdC	7.7 (1.3-21.1)	21.8 (4.7-53.3)	0.14
5-caCyt	8.0 (6.6-14.4)	12.2 (8.6-38.4)	0.24
5-fCyt	2.7 (1.9-3.8)	4.4 (3.0-6.2)	0.002*
5-hmUra	29.3 (15.7-47.0)	47.4 (28.3-87.1)	0.008*

Table S4. Urinary levels of DNA damage markers and active demethylation products of 5-methylcytosine in ETV6/RUNX1-negative ALL (I) and ETV6/RUNX1-positive ALL (II) group before therapy (A), 30 days after treatment (B) and six months after treatment (C).

		**5-hmdC**	**8-oxodG**	**5-hmCyt**	**5-mdC**	**5-caCyt**	**5-fCyt**	**5-hmUra**
I	**A**	12.4 (5.5-27.6)	2.8 (1.5-4.5)	12.5 (7.4-19.2)	12.3 (1.6-20.5)	7.1 (4.8-11.5)	2.5 (1.9-3.3)	27.3 (14.3-50.1)
	**B**	7.0 (4.3-14.3)	6.2 (4.0-8.1)	10.6 (6.0-13.9)	4.2 (3.4-10.6)	6.0 (5.2-7.9)	3.8 (2.7-4.8)	22.6 (14.9-33.2)
	**C**	5.3 (4.3-5.5)	7.3 (3.7-12.6)	5.6 (4.7-5.7)	2.1 (0.9-6.9)	-	2.1 (1.4-5.0)	15.2 (11.4-16.4)
**Control**		5.1 (3.6-8.0)	1.4 (1.0-1.9)	5.5 (4.5-6.9)	2.2 (0.7-4.3)	4.0 (3.0-4.7)	2.3 (1.6-3.1)	13.8 (10.7-18.6)
II	**A**	66.7 (28.9-187.5)	3.1 (1.9-4.9)	18.9 (10.0-34.2)	5.2 (3.1-6.1)	12.2 (8.7-38.4)	4.4 (2.9-6.7)	47.4 (29.1-89.3)
	**B**	12.3 (9.4-15.8)	4.5 (2.9-8.2)	10.4 (7.8-13.0)	3.9 (3.2-6.0)	9.4 (5.9-48.2)	4.6 (3.4-7.4)	31.6 (26.1-43.9)
	**C**	5.8 (5.8-5.8)	2.8 (2.8-2.8)	7.2 (5.5-8.8)	0.6 (0.6-0.6)	-	3.2 (2.5-4.0)	24.6 (17.5-31.6)

The results are presented as median values and interquartile ranges.

Figure S1. Expression of TDG protein in the leukocytes before starting the treatment and 33 days after the initiation of treatment.

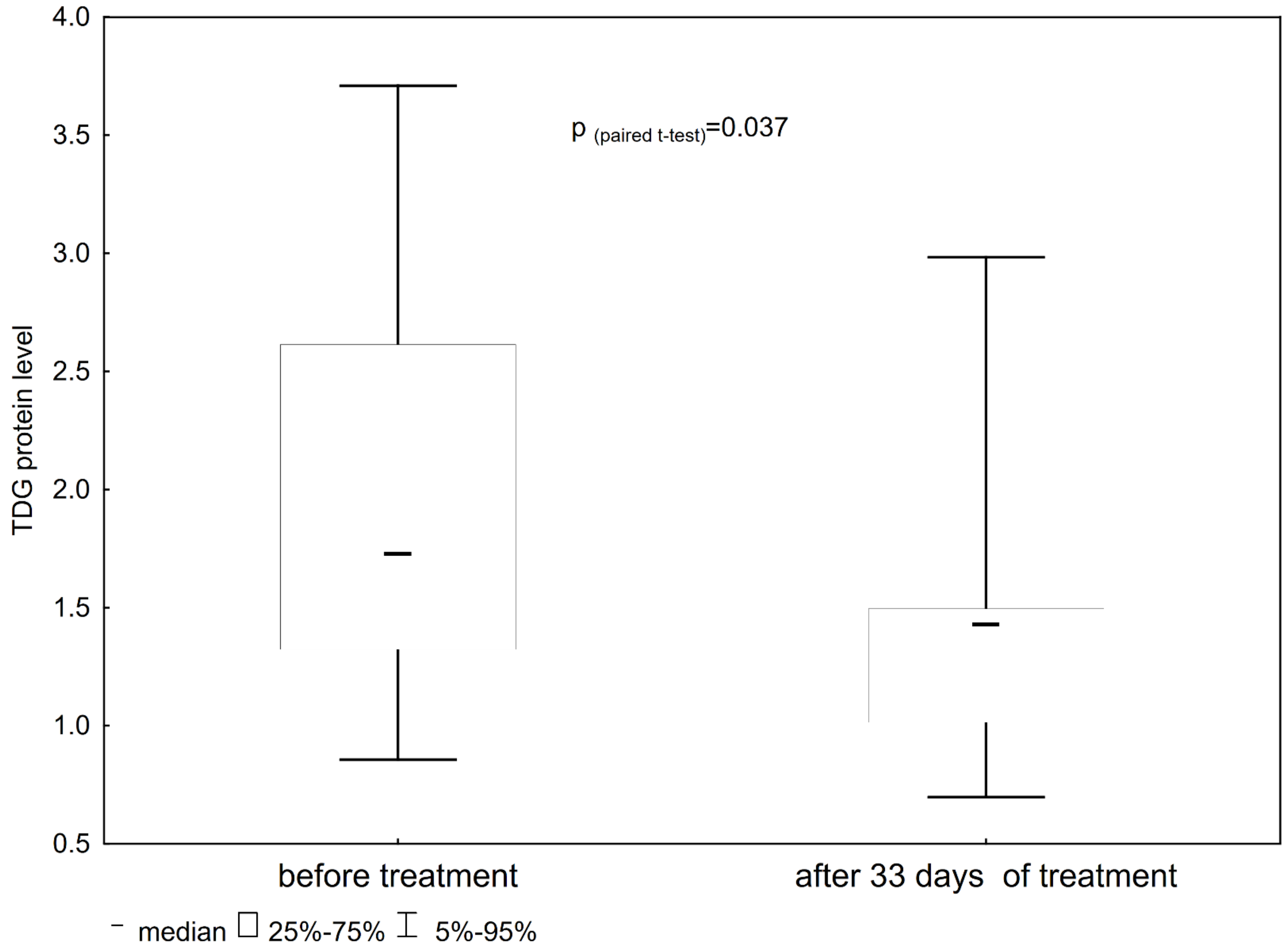

Figure S2. Correlations between TDG and TET proteins in CD45+/CD34+/CD19-/CD3- cells. Individual data points represent Box-Cox transformed values.

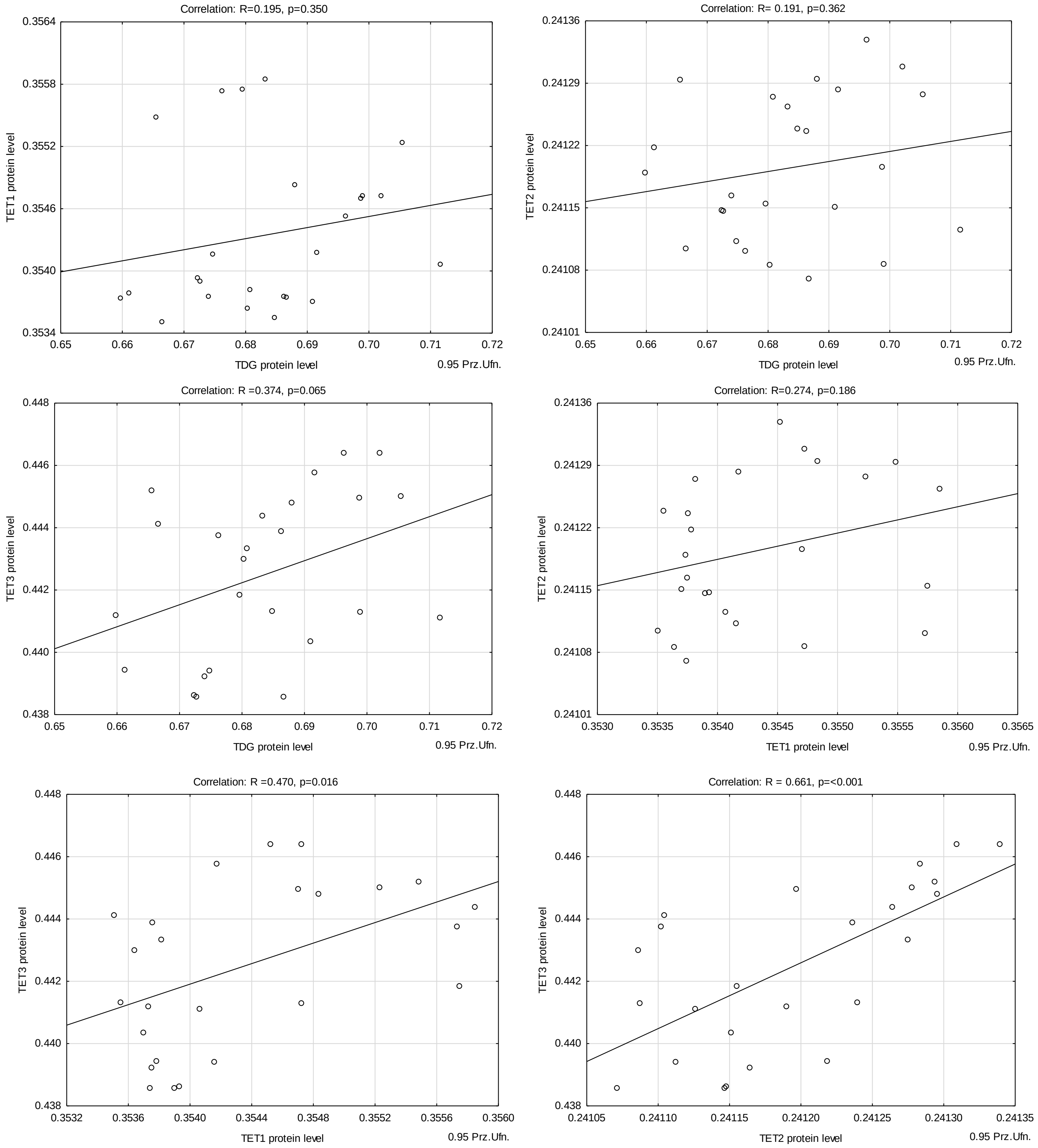

Figure S3. Correlations between TDG and TET proteins in granulocytes. Individual data points represent Box-Cox transformed values.

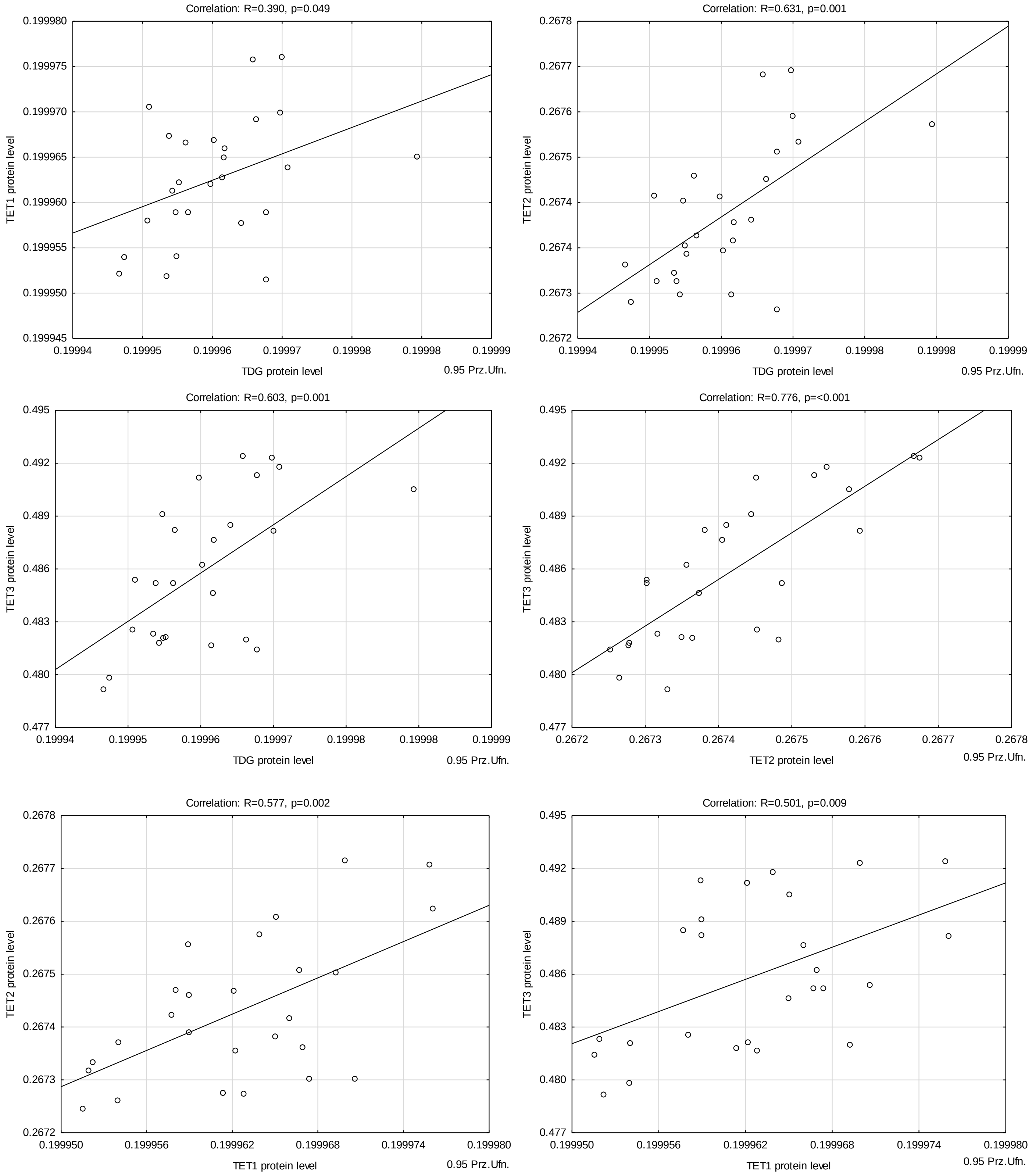

Figure S4. Correlations between TDG and TET proteins in monocytes. Individual data points represent Box-Cox transformed values.

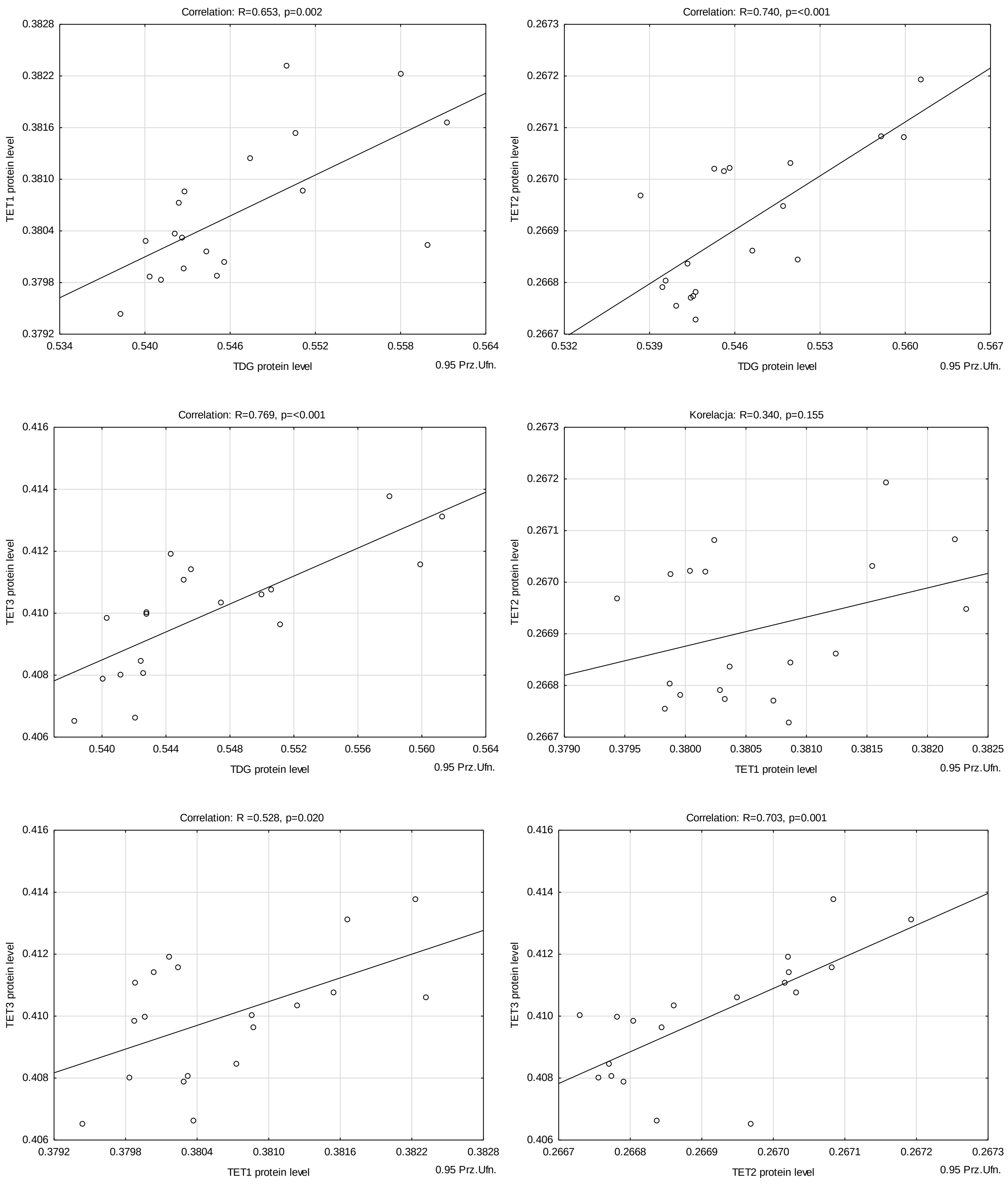

Figure S5. Correlations between TDG and TET proteins in lymphocytes. Individual data points represent Box-Cox transformed values.

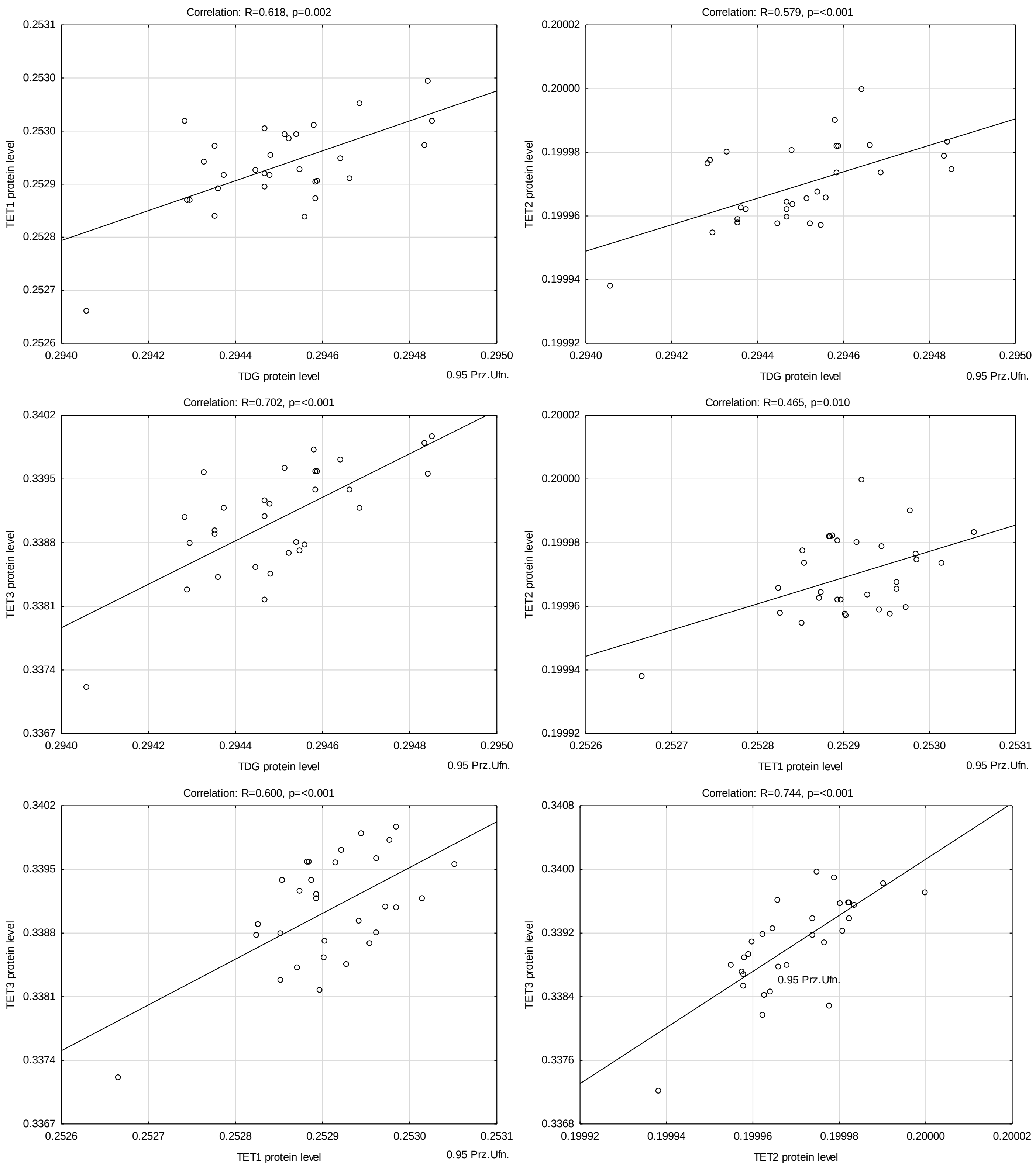

Figure S6. Correlations between the active DNA demethylation products and TET proteins in selected cell populations. Individual data points represent Box-Cox transformed values.

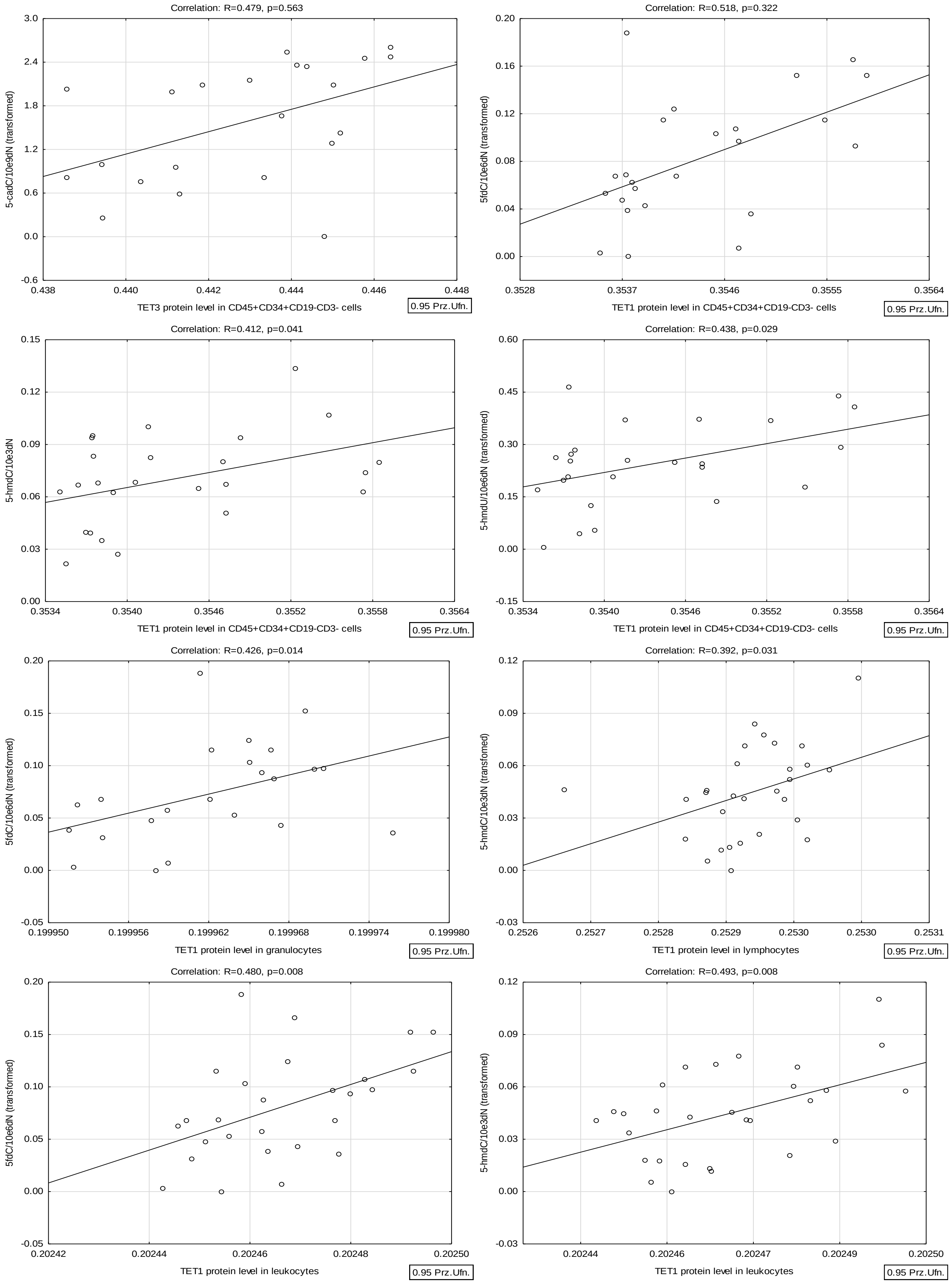

Methods

The determination of the epigenetic modifications and 8-oxodG levels in urine.

Two-dimensional ultra-performance liquid chromatography with tandem mass spectrometry (2D UPLC–MS/MS) was used for the epigenetic modification analysis of urine samples (with the exception of 5-hmUra). Urine samples were spiked with a mixture of internal standards at a 4:1 volumetric ratio. The 2D-UPLC−MS/MS system consists of a gradient pump and autosampler for one-dimensional chromatography, and a gradient pump and tandem quadrupole mass spectrometer with a UNISPRAY ion source was used for two-dimensional chromatography. Both systems were coupled with a column manager equipped with two programmable column heaters and two 2-position 6-port switching valves. The at-column dilution technique was used between the first and the second dimensions to improve the retention in the trap/transfer column. The sample molecules were then adsorbed to the packing material as very narrow bands that could be eluted with well-resolved, small-volume peaks. A diluting stream of water (0.5 mL/min) was pumped with a Waters 515 isocratic pump and mixed with the first-dimension column effluent using a UPLC low-dead-volume tee valve. The following columns were used: CORTECS UPLC T3 Column (1.6 µm, 3 mm × 150 mm) with a CORTECS T3 VanGuard precolumn (1.6 µm, 2.1 mm × 5 mm) for the first dimension, a Waters ACQUITY UPLC CSH C18 (1.7 µm, 2.1 mm × 100 mm) for the second dimension, and a Waters XSelect CSH C18 column (3.5 µm, 3 mm × 20 mm) as the trap/transfer column. The chromatographic system was operated in heart-cutting mode, which means that selected portions of effluent from the first dimension were loaded onto the trap/transfer column by 6-port valve switching, which served as an "injector" for the second dimension of the chromatography system. Mass spectrometric detection was conducted with a Waters Xevo TQ-S tandem quadrupole mass spectrometer equipped with a UniSpray ionization source. The following common detection parameters were used: source temperature, 150°C; nitrogen desolvation gas flow, 1000 L/h; nitrogen cone gas flow, 150 L/h; desolvation temperature, 500°C; and nebulizer gas pressure, 7 bar. Collision-induced dissociation was obtained with argon (6.0 at 3 × 10-6 bar pressure) as a collision gas. The instrument response to all compounds was optimized by the infusion of 10 µM genuine compounds dissolved in water (10 µL/minute) in the mobile phase A stream via the mass spectrometer fluidics system operating in the "mixed" mode using MassLynx 4.1 software IntelliStart feature. The quantitative and qualitative transition patterns and the specific settings of the detector are summarized in Table S5. The chromatographic system was operated with MassLynx 4.1 software from Waters. Quantitative analyses were performed using the TargetLynx application. All samples were analyzed with three to six technical replicates. Due to the low sensitivity of the method used, the level of 5-hmUra was determined by high-performance liquid chromatography for pre-purification followed by gas chromatography with isotope dilution mass spectrometric detection (LC/GC–MS), as previously described [1].

Isolation of DNA and the determination of the epigenetic modifications and 8-oxodG in DNA isolates

Leukocytes were isolated from heparinized blood samples with Histopaque 1119 (Sigma) solution, according to the manufacturer's instructions, and stored at −80°C until analysis. The analyses were performed using a method described earlier by Gackowski et al. with some modifications [2]. Briefly, a pellet of frozen cells was dispersed in ice-cold buffer B (Tris-HCl (10 mmol/L), Na_2EDTA (5 mmol/L) and deferoxamine mesylate (0.15 mmol/L), pH 8.0). SDS solution was added (to a final concentration of 0.5%), and the mixture was gently mixed using a polypropylene Pasteur pipette. The samples were incubated at 37 °C for 30 minutes. Proteinase K was added to a final concentration of 2.5 mg/mL and incubated at 37 °C for 1.5 h. The mixture was cooled to 4 °C, transferred to a centrifuge tube with phenol:chloroform:isoamyl alcohol (25:24:1), and vortexed vigorously. After extraction, the aqueous phase was treated with a chloroform:isoamyl alcohol mixture (24:1). The supernatant was treated with two volumes of cold absolute ethanol to precipitate high molecular weight nucleic acids. The precipitate was removed with a plastic spatula, washed with 70% (v/v) ethanol and dissolved in Milli-Q grade deionized water. The samples were mixed with 200 mM ammonium acetate containing 0.2 mM $ZnCl_2$, pH 4.6 (1:1 v/v). Nuclease P1 (1 U) and tetrahydrouridine (10 µg/sample) were added to the mixture and incubated at 37°C for 1 h. Subsequently, 13 µL and 15 µL of 10% (v/v) NH_4OH (for the remaining nucleic acids and ultrafiltrate, respectively) and 1.3 U of alkaline phosphatase were added to each sample and incubated for 1 h at 37 °C. Finally, all the

hydrolysates were ultra-filtered prior to injection. The DNA hydrolysates were spiked with a mixture of internal standards at a volumetric ratio of 4:1 to a final concentration of 50 fmol/µL: [D_3]-5-hmdC, [$^{13}C_{10}$, $^{15}N_2$]-5-fdC, [$^{13}C_{10}$, $^{15}N_2$]-5-cadC, and [$^{13}C_{10}$, $^{15}N_2$]-5-hmdU, [^{13}C, $^{15}N_2$]-2'-deoxyuridine, and [$^{15}N_5$]-8-oxo-7,8-dihydro-2'-deoxyguanosine. Chromatographic separation was performed with a Waters ACQUITY 2D-UPLC system with a photodiode array detector for the first dimension of the 2D-chromatography (used for quantification of the unmodified deoxynucleosides and 5-mdC) and a Xevo TQ-S tandem quadrupole mass spectrometer (used for the second dimension of the 2D-chromatography to analyze the compounds from the first dimension in positive mode, namely, 5-hmdC and 8-oxodG, to assure better ionization at higher acetic acid concentrations). The at-column dilution technique was used between the first and second dimensions to improve the retention of the trap/transfer column. The following columns used: a Waters CORTECS T3 column (150 mm×3 mm, 1.6 µm) with a precolumn for the first dimension, a Waters XSelect C18 CSH (100 mm×2.1 mm, 1.7 µm) for the second dimension and a Waters XSelect C18 CSH (20 mm×3 mm, 3.5 µm) column as a trap/transfer column. The chromatographic system was operated in heart-cutting mode, indicating that selected fractions of the effluent from the first dimension were loaded onto the trap/transfer column by 6-port valve switching, which served as the “injector” for the second dimension of the 2D-chromatography process. The flow rate for the first dimension was 0.5 mL/minute, and the injection volume was 2 µL. Separation was performed with a gradient elution for 10 minutes using a mobile phase of 0.05% acetate (A) and acetonitrile (B) (0.7-5% B for 5 minutes, column washing with 30% acetonitrile and re-equilibration with 99% A for 3.6 minutes). The flow rate for the second dimension was 0.3 mL/minute. The separation was performed with a gradient elution for 10 minutes using a mobile phase of 0.01% acetate (A) and methanol (B) (1-50% B for 4 minutes, isocratic flow of 50% B for 1.5 minutes, and re-equilibration with 99% A until the next injection). All samples were analyzed with three to five technical replicates, of which the technical mean was used for further calculation. Mass spectrometric detection was performed using a Waters Xevo TQ-S tandem quadrupole mass spectrometer equipped with an electrospray ionization source. Collision-induced dissociation was obtained using argon 6.0 at 3×10^{-6} bar pressure as the collision gas. Transition patterns for all the analyzed compounds and the specific detector settings were determined using the MassLynx 4.1 IntelliStart feature set in quantitative mode to ensure the best signal-to-noise ratio and a resolution of 1 at MS1 and 0.75 at MS2.

Determination of ascorbate level in blood plasma by UPLC-UV

Sample preparation

To stabilize ascorbate and to precipitate proteins, 200-µL aliquots of freshly prepared or partially thawed plasma were mixed with 200 µL of precooled 10% (w/v) meta-phosphoric acid (MPA, Sigma-Aldrich, Munich, Germany) containing uracil (50 µM, Sigma-Aldrich) as an internal standard. The samples were maintained on ice for 40 minutes and then diluted with 200 µL of Milli-Q grade deionized water (Merck Millipore), vortexed and centrifuged at 25,155×g for 15 minutes at 4 °C. The supernatants (200 µL) were purified by ultrafiltration using AcroPrep Advance 96-well filter plates 10 K (Pall) and injected into a Waters ACQUITY ultra-performance liquid chromatographic (UPLC) system. The method was validated with the reference material from Chromsystems.

Chromatography

The UPLC system consisted of a binary solvent manager, sample manager, column manager and photodiode array detector, all from Waters. The samples were separated on a Waters ACQUITY UPLC HSS T3 column (1.8 µm, 150 mm × 2.1 mm) with a Van Guard HSS T3 1.8-µm precolumn at a flow rate of 0.25 mL/minute and a 2-µL injection volume. Ammonium formate (10 mM, pH 3.1, Fluka) and acetonitrile (Sigma-Aldrich) were used as solvents A and B, respectively. The following program was used for the ascorbate elution: 0–0.1 minutes, 99% A, 1% B; 0.1–2.2 minutes, 97% A; 2.2–4.0 min, linear gradient to 90% A; 4.0–4.5 minutes, 90% A; and 4.5–6.0 minutes, 99% A. The column thermostat was set at 10 °C. The effluent was monitored with a photodiode array detector set at 245 nm and analyzed with Empower software.

Gene expression analysis

RNA isolation was performed using the PAXgene blood RNA kit (Qiagen) following standard procedures. The concentration and purity of the RNA aliquots were verified spectrophotometrically with a NanoDrop 2000 spectrophotometer (Thermo Scientific). The A_{260}/A_{280} ratio was used as an indicator of protein contamination, and the A_{260}/A_{230} ratio was used as a measure of contamination with polysaccharides, phenol and/or chaotropic salts. The quality and integrity of the total RNA were assessed by visualization of the 28S/18S/5.8*S rRNA* band pattern in a 1.2% agarose gel. Nondenaturing electrophoresis was carried out at 95 V for 20 minutes in TBE buffer (Tris, boric acid and EDTA). The gel was stained with ethidium bromide or SimplySafe and visualized using a G:BOX EF gel documentation system (Syngene).

The samples with RNA concentrations greater than 50 ng/µL were qualified for further analysis. A total of 0.5 micrograms of total RNA from each sample (in a 20-µl volume) was used for cDNA synthesis by reverse transcription with a high-capacity cDNA reverse transcription kit (Applied Biosystems, catalog no. 43-688-14) according to the manufacturer's instructions. The reaction was carried out with a Mastercycler nexus gradient thermocycler (Eppendorf). To exclude contamination with genomic DNA, reverse transcriptase reaction also included a negative control. cDNA was either immediately used for qPCR or stored at −20°C.

Gene transcripts were analyzed by relative quantitative RT-PCR (RT-qPCR) with relevant primers and short hydrolysis probes substituted with locked nucleic acids from the Universal Probe Library (UPL, Roche). The probes were labeled with fluorescein (FAM) at the 5'-end and with a dark quencher dye at the 3'-end. The expression of all target genes was normalized against the same set of four selected reference genes—HMBS (GeneID: 3145), TBP (GeneID: 6908), ACTB (GeneID: 60) and G6PD (GeneID: 2539)—using a UPL ready assay from Roche. Each real-time PCR mix (in a 20-µl volume) was prepared from cDNA following the standard procedures for the LightCycler 480 Probe Master Mix (Roche), as provided with the reagent set. The reactions were carried out on 96-well plates. In addition to the proper samples, each plate also included a no-template control and no-RT control. Quantitative real-time PCR was carried out on a LightCycler 480 II using the following cycling parameters: 10 s at 95°C, followed by 45 repeats of 10 s each at 95°C, 30 s at 58°C, and finally, 1 s at 72°C in the acquisition mode (with the parameters of wavelength excitation and detection set to 465 nm and 510 nm, respectively). The reaction for each gene was standardized against a standard curve to estimate amplification efficiency. The standardization procedure included the preparation of 10-fold serial dilutions with a controlled relative amount of targeted template. The efficiency of amplification was assessed based on the slope of the standard curve. Standard dilutions were amplified in separate wells but within the same run.

Table S5. Primers and short hydrolysis probes used for the target gene mRNA expression analysis.

Gene	Forward primer sequence	Reverse primer sequence	UPL (Roche)
TET1	5'-TCTGTTGTTGTGCCTCTGGA-3'	5'-GCCTTTAAAACTTTGGGCTTC-3'	#57
TET2	5'-GCCTTTGCTCCTGTTGAGTT-3'	5'-ACAAGGCTGCCCTCTAGTTG-3'	#38
TET3	5'-CACTCCGGAGAAGATCAAGC-3'	5'-GGACAATCCACCCTTCAGAG-3'	#1
TDG	5'-GAATGGAAGCGGAGAACG-3'	5'-TTGCTGTTCATTCACAACTGC-3'	#41

Protein expression analysis

Because of the lack of commercially available anti-TETs (Tet1, Tet2, Tet3) antibodies recommended for direct flow cytometry method, it was decided to use an indirect staining consisting of 2 steps involving labeling with unconjugated primary and compatible fluorochrome-conjugated secondary antibodies. Both type of antibodies were titrated.

This process enables the determination of the antibody amount and concentration, resulting in the highest possible signal from the positive population and the lowest signal from the negative population. Nonspecific antibody-binding was eliminated by optimizing the amount and concentration of the antibody [3].

The following antibodies were used: 1) rabbit polyclonal anti-Tet1 antibody (Cat. No. ab121587), Abcam; 2) goat polyclonal anti-Tet2 antibody (Cat. No. ab99432), Abcam; 3) rabbit polyclonal anti-Tet3 antibody (Cat. No. ab139311).

The secondary antibodies were selected based on the primary antibodies and conjugated with a fluorochrome: 1) donkey F(ab')2 anti-rabbit IgG H&L (Alexa Fluor® 488) (Cat. No. ab181346, Abcam); 2) rabbit F(ab')2 anti-goat IgG H&L (Alexa Fluor® 647) (Cat. No. ab169347, Abcam).

For titration, the following primary antibody amounts were used: 0.5 µl, 0.6 µl, 0.8 µl, 1 µl, and 1.2 µl. The secondary antibody dilutions were 1:400 (µl), 1:1000 (µl), 1:2000 (µl), and 1:4000 (µl). In the first step, the secondary antibody was titrated, and a dilution of 1:1000 was selected. Then, the primary antibodies were titrated, and 1 µl was chosen. Perm/Wash Buffer from BD Cytofix/Cytoperm™- (BD Biosciences) was used for the dilutions.

Blood specimens were collected by venipuncture into tubes containing tripotassium EDTA and the cellular surface antigen-stabilizing agent TransFix. Before the staining procedure was performed, the cells were counted using a LUNA-II automated cell counter. This number was important for ensuring the correct amount of antibodies was added. For the purposes of our experiment, a volume of blood containing 100 000 cells was added to a 5-ml round-bottom tube and suspended in PBS to obtain a final volume of 100 µl.

Fluorochrome-labeled monoclonal antibodies: PE-anti-human CD56/CD16, PerCP-anti-human CD14, APC-H7-anti-human CD19, BV42-anti-human CD45, V500-anti-human CD3, and PE-Cy7-anti-human CD34 (BD Biosciences, Franklin Laker, New Jersey, USA) were added to each of the tubes according to the scheme shown in Table S6 with an amount recommended by the manufacturer.

Table S6. Panel of surface labelling markers.

NUMBER OF TUBES	MONOCLONAL ANTIBODIES
**1.**	CD45BV421/CD34PE-Cy7
**2.**	CD45BV421/CD56+CD16PE/CD14PerCP/CD19APC-H7/CD3V500/CD34PE-Cy7
**3.**	CD45BV421/CD56+CD16PE/CD14PerCP/CD19APC-H7/CD3V500/CD34PE-Cy7
**4.**	CD45BV421/CD56+CD16PE/CD14PerCP/CD19APC-H7/CD3V500/CD34PE-Cy7

The samples were incubated at room temperature in the dark for 30 minutes. Then, the erythrocytes were lysed with 2 ml of FACS lysing solution (BD Biosciences) and incubated for 15 minutes under the same conditions as the previous incubation. After two washes with PBS and centrifugation at 500 × g for 5 minutes, the cell pellets were resuspended in 500 µl of BD Cytofix/Cytoperm™ solution (BD Biosciences) to fix and permeabilize the cells. The cells were incubated in the dark for 20 minutes at room temperature. Then, the cells were centrifuged at 500 x g for 5 minutes again. Cell pellets were resuspended in freshly prepared Perm/Wash Buffer and incubated for 10 minutes in the dark. Then, the cells were centrifuged, the supernatant was removed, and the pellets were treated by primary antibodies to the scheme shown in Table S7.

Table S7. Panel of intracellular labelling.

NUMBER OF TUBES	PRIMARY ANTIBODIES	SECONDARY ANTIBODIES
**1.**	Perm wash buffer	Perm wash buffer
**2.**	Perm wash buffer	Donkey anti-rabbit Alexa Fluor 488/Rabbit anti-goat Alexa Fluor 647
**3.**	Rabbit polyclonal anti-human TET-1	Donkey anti-rabbit Alexa Fluor 488
**4.**	Rabbit polyclonal anti-human TET-3/goat polyclonal anti human TET-2/	Donkey anti-rabbit Alexa Fluor 488/rabbit anti-goat Alexa Fluor 647

The secondary antibody solution of donkey anti-rabbit Alexa Fluor 488 or rabbit anti-goat Alexa Fluor 647 (Abcam) was prepared and 100 µL of the 1000-fold diluted antibody was added to the samples. One hundred microliters of Perm wash buffer was added to tube no. 1. Sample no. 2, which included only surface and secondary antibodies, was a control for the investigated proteins. The tubes were incubated at room temperature for 30 minutes in the dark. Then, 1 mL of Perm wash buffer was added per tube and incubated for 10 minutes at room temperature in the dark. Next, the samples were centrifuged (500 × g for 5 minutes), and the supernatant was removed by pipette. The cell pellets were resuspended in 250 µL of PBS and then analyzed using a three-laser BD FACS Canto II flow cytometer (BD Biosciences) [4]. The instrument was set up using a BD Cytometer Setup and Tracking beads. Data on 30,000 cells were acquired with BD FACSDiva software and analyzed with FlowJo 7.5.5 software (Tree Star Inc., Ashland, Oregon, USA).

Cell doublets were rejected by setting the gate for single cells in the plot linear forward-scatter area (FSC-A) vs. linear forward-scatter height (FSC-H). The leukocytes (granulocytes, monocytes and

lymphocytes, and atypical cells) were gated on the basis of their morphological features, as measured by linear side-scatter area (SSC-A) and CD45 antigen expression (logarithmic CD45 Brilliant Violet 421-H). For each population, the fluorescence was detected separately and plotted in histogram mode: counts vs. logarithmic Alexa Fluor 488-H and counts vs. logarithmic Alexa Fluor 647-H. The geometric mean of the fluorescence intensity (GMFI) was calculated for the total cell population in each channel. The expression of the investigated proteins was calculated as the geometric mean fluorescence intensity in the test sample as a fold change over the negative control (tube no. 2) (ratio= GMFI test tube/GMFI control tube).

Supplementary References

1. Rozalski R, Gackowski D, Siomek-Gorecka A, et al. Urinary 5-hydroxymethyluracil and 8-oxo-7,8-dihydroguanine as potential biomarkers in patients with colorectal cancer. Biomarkers. 2015;20(5):287-291.
2. Gackowski D, Starczak M, Zarakowska E, et al. Accurate, Direct, and High-Throughput Analyses of a Broad Spectrum of Endogenously Generated DNA Base Modifications with Isotope-Dilution Two-Dimensional Ultraperformance Liquid Chromatography with Tandem Mass Spectrometry: Possible Clinical Implication. Anal Chem. 2016;88(24):12128-12136.
3. Uhlen M, Oksvold P, Fagerberg L, et al. Towards a knowledge-based Human Protein Atlas. Nat Biotechnol. 2010;28(12):1248-1250.
4. Dagur PK, McCoy JP, Jr. Collection, Storage, and Preparation of Human Blood Cells. Curr Protoc Cytom. 2015;73(5 1 1-5 1 16.

Table S8. Transition patterns and specific detector settings for all compounds analyzed by 2D UPLC–MS/MS in urine.

compound name		ionization mode	nominal molecular mass (Da)	pseudo-molecular ion formulation	nominal parent ion (Da)	nominal daughter ion (Da)	capillary (kV)	cone (V)	collision (eV)
**5-hydroxymethylcytosine**	quantifier	UniSpray+	141	$[M+H]^+$	142	81	1.3	40	18
	qualifier	UniSpray+	141	$[M+H]^+$	142	124	1.3	40	12
**[D_3]-5-hydroxymethylcytosine**	quantifier	UniSpray+	144	$[(M+3)+H]^+$	145	84	1.3	40	18
	qualifier	UniSpray+	144	$[(M+3)+H]^+$	145	127	1.3	40	12
**5-hydroxymethyl-2'-deoxycytidine**	quantifier	UniSpray+	257	$[M+H]^+$	258	124	1.3	35	22
	qualifier	UniSpray+	257	$[M+H]^+$	258	142	1.3	35	10
**[D_3]-5-hydroxymethyl-2'-deoxycytidine**	quantifier	UniSpray+	260	$[(M+3)+H]^+$	261	127	1.3	35	22
	qualifier	UniSpray+	260	$[(M+3)+H]^+$	261	145	1.3	35	10
**5-carboxylcytosine**	quantifier	UniSpray-	155	$[M-H]^-$	154	67	1.3	60	12
	qualifier	UniSpray-	155	$[M-H]^-$	154	110	1.3	60	12
**[$^{13}C_{10}$, $^{15}N_2$]-5-carboxylcytosine**	quantifier	UniSpray-	162	$[(M+7)-H]^-$	161	71	1.3	60	12
	qualifier	UniSpray-	162	$[(M+7)-H]^-$	161	116	1.3	60	12
**8-oxo-2’-deoxyguanosine**	quantifier	UniSpray+	283	$[M+H]^+$	284	168	1.3	30	18
	qualifier	UniSpray+	283	$[M+H]^+$	284	140	1.3	30	18
**[$^{15}N_5$]-8-oxo-2'-deoxyguanosine**	quantifier	UniSpray+	288	(M+5)+H+	289	173	1.3	20	15
	qualifier	UniSpray+	288	(M+5)+H+	289	145	1.3	20	15
**5-formylcytosine**	quantifier	UniSpray+	139	$[M-H]^-$	140	97	1.3	40	15
	qualifier	UniSpray+	139	$[M-H]^-$	140	70	1.3	40	15
**[$^{13}C_{10}$, $^{15}N_2$]-5-formylcytosine**	quantifier	UniSpray+	146	$[(M+7)-H]^-$	147	102	1.3	40	15
	qualifier	UniSpray+	146	$[(M+7)-H]^-$	147	73	1.3	40	15
**5-methyl-2’-deoxycytidine**	quantifier	UniSpray+	241	$[M-H]^-$	242	126	1.3	30	12
	qualifier	UniSpray+	241	$[M-H]^-$	242	224	1.3	30	12
**[$^{13}C_{10}$, $^{15}N_2$]-5-methyl2’-deoxycytidine**	quantifier	UniSpray+	253	$[(M+12)-H]^-$	254	133	1.3	30	12
	qualifier	UniSpray+	241	$[(M+12)-H]^-$	254	236	1.3	30	12
